# An mRNA vaccine for pancreatic cancer designed by applying *in silico* immunoinformatics and reverse vaccinology approaches

**DOI:** 10.1371/journal.pone.0305413

**Published:** 2024-07-08

**Authors:** Md. Habib Ullah Masum, Shah Wajed, Md. Imam Hossain, Nusrat Rahman Moumi, Asma Talukder, Md. Mijanur Rahman

**Affiliations:** 1 Department of Microbiology, Noakhali Science and Technology University, Noakhali, Bangladesh; 2 Microbiology, Cancer and Bioinformatics Research Group, Noakhali Science and Technology University, Noakhali, Bangladesh; 3 Infectiology: Biology of Infectious Diseases, Universite Paris-Saclay, Gif-sur-Yvette, France; 4 Medical Sciences, University of Central Lancashire, Preston, Lancashire, United Kingdom; 5 Department of Biotechnology and Genetic Engineering, Noakhali Science and Technology University, Noakhali, Bangladesh; 6 School of Pharmacy and Medical Sciences, and Menzies Health Institute Queensland, Griffith University, Brisbane, Queensland, Australia; The Islamia University of Bahawalpur Pakistan, PAKISTAN

## Abstract

Pancreatic ductal adenocarcinoma is the most prevalent pancreatic cancer, which is considered a significant global health concern. Chemotherapy and surgery are the mainstays of current pancreatic cancer treatments; however, a few cases are suitable for surgery, and most of the cases will experience recurrent episodes. Compared to DNA or peptide vaccines, mRNA vaccines for pancreatic cancer have more promise because of their delivery, enhanced immune responses, and lower proneness to mutation. We constructed an mRNA vaccine by analyzing S100 family proteins, which are all major activators of receptors for advanced glycation end products. We applied immunoinformatic approaches, including physicochemical properties analysis, structural prediction and validation, molecular docking study, *in silico* cloning, and immune simulations. The designed mRNA vaccine was estimated to have a molecular weight of 165023.50 Da and was highly soluble (grand average of hydropathicity of -0.440). In the structural assessment, the vaccine seemed to be a well-stable and functioning protein (Z score of -8.94). Also, the docking analysis suggested that the vaccine had a high affinity for TLR-2 and TLR-4 receptors. Additionally, the molecular mechanics with generalized Born and surface area solvation analysis of the "Vaccine—TLR-2" (-141.07 kcal/mol) and "Vaccine—TLR-4" (-271.72 kcal/mol) complexes also suggests a strong binding affinity for the receptors. Codon optimization also provided a high expression level with a GC content of 47.04% and a codon adaptation index score 1.0. The appearance of memory B-cells and T-cells was also observed over a while, with an increased level of helper T-cells and immunoglobulins (IgM and IgG). Moreover, the minimum free energy of the mRNA vaccine was predicted at -1760.00 kcal/mol, indicating the stability of the vaccine following its entry, transcription, and expression. This hypothetical vaccine offers a groundbreaking tool for future research and therapeutic development of pancreatic cancer.

## 1. Introduction

Pancreatic ductal adenocarcinoma (PDAC), which constitutes 90% of pancreatic cancer (PC), is the fourth most prevalent cause of cancer-related mortality globally [1]. A study suggests that by 2030, the number of deaths in the US from PC will surpass that from breast, prostate, and colorectal cancer combined, partly as a result of improvements in the treatment of other cancers and an aging population [2]. In a recent study by the American Cancer Society (ACS) [3], the overall 5-year survival rate for PC is dramatically low, estimated at around 12%. The poor survival rate may be attributed to several factors, one of which is the late stage at which most patients are diagnosed [4]. Identifying the early stage of PC is challenging as there is a scarcity of symptoms and biomarkers that are precise to it [5]. In most cases, patients have already reached an incurable advanced stage by the time they exhibit symptoms and are diagnosed [5]. PDAC is primarily treated with chemotherapy and surgery, but because of distant metastasis at the diagnosis stage, the eligibility for surgical intervention is confined to a range of 15%-20% of patients [5]. Even when surgery is an alternative, approximately three-quarters of patients will have a recurrence within two years of surgery. Another developing approach for PC is neoadjuvant therapy, particularly for borderline and locally advanced unresectable cases [6]. Unfortunately, the randomized trial for neoadjuvant chemoradiotherapy in PC had to be stopped prematurely due to insufficient patient enrollment and outcomes that did not show statistical significance [7]. Based on different clinical and preclinical studies, mRNA-based therapeutics were found equal to or more effective than DNA or peptide platforms in delivering cancer vaccines [8]. The mRNA approach is adaptable and has effectively been employed in various vaccine delivery strategies, including systemic, subcutaneous, intramuscular, and in situ methods, as well as in genetically modifying dendritic cell-based vaccines and developing chimeric antigen receptor (CAR) T-cell therapies [8]. Also, mRNA-driven cancer vaccines encode complete cancer antigens, overcoming human leukocyte antigen restrictions for a wider immune reaction and remaining mutation-free due to mRNA’s inability to integrate into chromosomes [9].

Recent studies suggest that receptor for advanced glycation endproducts (RAGE) plays a significant role in the advancement of PC and might serve as a promising target for therapeutic interventions [10]. However, RAGE may be triggered by many members of the S100 protein family alongside being activated by other ligands [11]. S100 proteins are of notable importance in developing vaccines for PC, considering their unique attributes and implications for the progression of the disease [11, 12]. These proteins frequently appear in higher concentrations in PC tissues, serving as robust biomarkers for disease detection and prognosis. Their abnormal expression identifies malignant cells and contributes to their prospective targets for immunotherapeutic interventions [11, 12]. This offers the potential to develop vaccines that aim to stimulate an immune response against malignant tissues while excluding healthy ones. The S100 protein family consists of 21 members, which have a significant degree of structural similarity and regulate cellular responses by serving as both intracellular calcium (Ca^2+^) sensors and extracellular factors [13]. Among the different proteins, S100A4 is considered a risk factor for PC [14, 15], which does not express in normal tissues but is highly expressed in PC cells and related to the tumor-node-metastasis (TNM) staging and tumor size in PC [12]. S100A6 is a biomarker in PC lesions restricted to the nuclei in PC cells but not in the noncancerous tissues [16, 17]. S100A8 and S100A9 are two overexpressed proteins that are potential inflammatory mediators occurring in PDAC immunosuppression and suppress T-cell activation [18]. S100A11 is another potential gene therapy target, overexpressed in PC cells, and facilitates the PDAC interstitium and promotes PDAC growth [19].

In this study, we aimed to design a novel mRNA vaccine targeting five members of the S100 family protein, S100-A4, S100-A6, S100-A8, S100-A9, and S100-A11, which consists of cytotoxic T lymphocyte (CTL), helper T lymphocyte (HTL), linear B-cell epitopes derived from the selected proteins. With a combination of highly immunogenic adjuvants such as Heparin-binding hemagglutinin (HBHA) and five additional linkers, namely EAAAK, AYY, AK, KFER, and GPGPG, we designed the vaccine construct applying the immunoinformatic and computational strategies.

## 2. Methods

### 2.1 Retrieval of protein sequence

**Fig 1:**
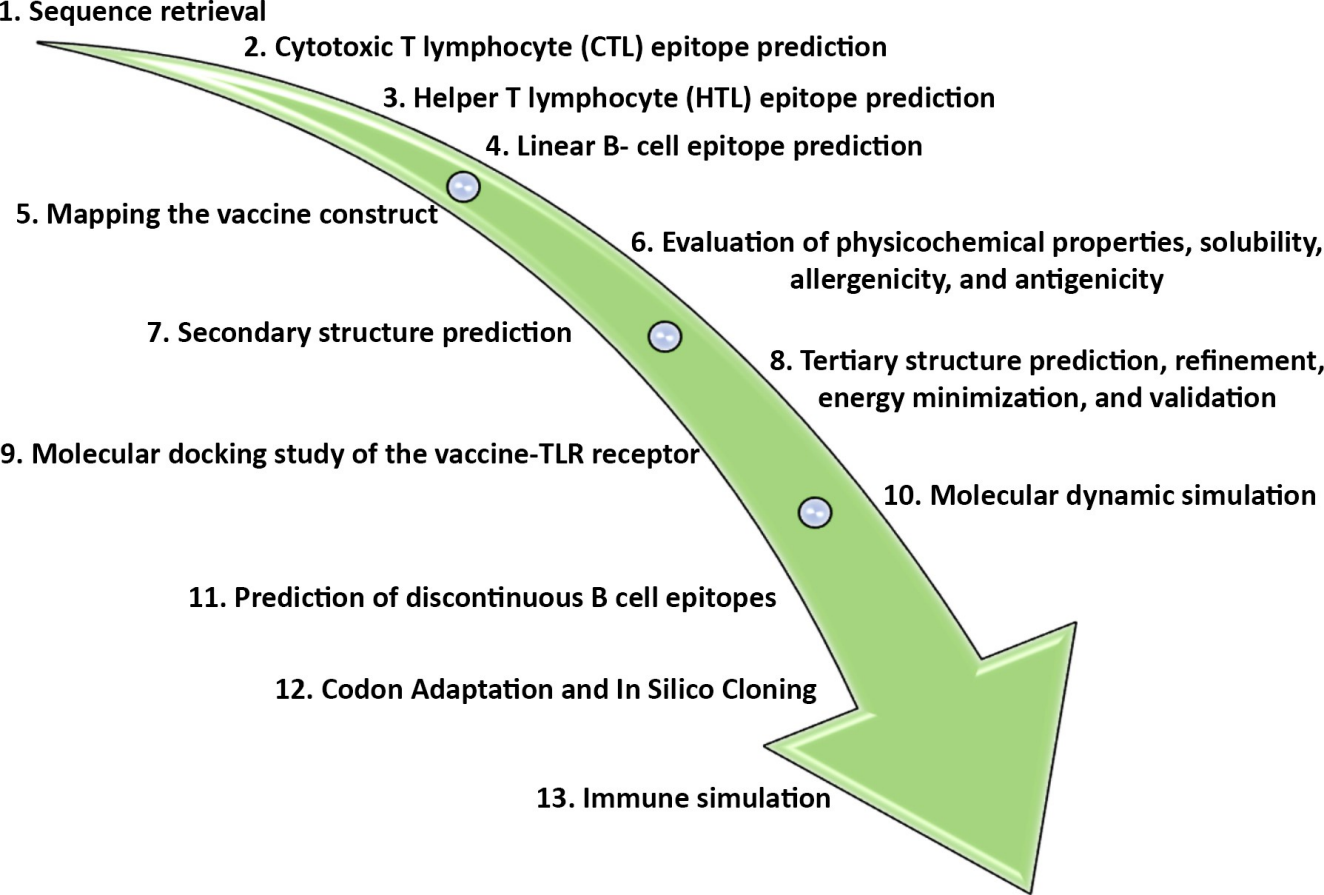
An overview of the study.

Amino acid sequences of the following proteins, S100-A4 (accession number: P26447.1), S100-A6 (accession number: P06703.1), S100-A8 (accession number: P05109.1), S100-A9 (accession number: P06702.1), S100-A11 (accession number: P31949.2) were retrieved from National Center for Biotechnology Information (NCBI) (https://www.ncbi.nlm.nih.gov/) protein database and saved in FASTA format. The FASTA sequences were subsequently utilized for vaccine development. The summary of the study is depicted in **Fig 1**.

### 2.2 Cytotoxic T lymphocyte (CTL) epitope prediction

The Immune Epitope Database (IEDB) server (http://tools.iedb.org/mhci/) was applied to predict CTL epitopes from the intended protein sequences [20–30]. The server predicts CTL epitopes from a protein sequence based on affinity for major histocompatibility complex- I (MHC-I), the transportation efficiency (TAP), and the cleavage of the proteasome [21, 31]. We choose 12 MHC- I binding alleles, including HLA-A1, HLA-A2, HLA-A3, HLA-A24, HLA-A26, HLA-B7, HLA-B8, HLA-B27, HLA-B39, HLA-B44, HLA-B58, and HLA-B62 and applied the IEDB recommended method (NetMHCpan 4.1 EL) for this prediction. The predicted epitopes were further assessed for immunogenicity, antigenicity and toxicity through different validation tools like the IEDB class I immunogenicity (http://tools.iedb.org/immunogenicity/) [32, 33], Vaxijen v2.0 (http://www.ddg-pharmfac.net/vaxijen/VaxiJen/VaxiJen.html) [34] and ToxinPred Server (http://crdd.osdd.net/raghava/toxinpred/) [35, 36], respectively. The prediction threshold value was set to 0.5 for both Vaxijen v2.0 and ToxinPred server. However, the Vaxijen v2.0 has a prediction accuracy of 70% to 89%, while ToxinPred has a prediction accuracy of 94.50%.

### 2.3 Helper T lymphocyte (HTL) epitope prediction

The HTLs are recognized as having a variety of functions, including regulating T- and B-cells, identifying antigens through Major histocompatibility complex-II (MHC-II) on antigen-presenting cell (APC), helping in T-cell-mediated immunity, and so on [37]. Therefore, HTLs are crucial in developing adaptive immune responses [37]. Furthermore, the HTL epitope is critical for efficient vaccine development since vaccine antigen (Ag) is processed to be delivered via MHC-II [38, 39]. The IEDB MHC-II binding server was applied to screen HTL epitopes from intended protein sequences. The program was run by IEDB recommended method with 13 different MHC-II alleles, including HLA-DRB1-0101, HLA-DRB1-0301, HLA-DRB1-0401, HLA-DRB1-0701, HLA-DRB1-0801, HLA-DRB1-0901, HLA-DRB1-1001, HLA-DRB1-1101, HLA-DRB1-1201, HLA-DRB1-1301, HLA-DRB1-1401, HLA-DRB1-1501, and HLA-DRB1-1601. All the selected epitopes were further applied for the *in silico* assessment of interleukin-10 (IL-10) and interferon-gamma (IFN-γ) through IL-10Pred (http://crdd.osdd.net/raghava/IL-10pred/) and IFNepitope (http://crdd.osdd.net/raghava/ifnepitope/predict.php), respectively. However, the threshold value was set to -0.3 while predicting with IL-10pred, but it was set to 0.5 in the case of INFepitope. Additionally, the antigenicity and toxicity were also evaluated by Vaxijen v2.0 [34] and ToxinPred server [35, 36] with a threshold value of 0.5.

### 2.4 B-cell (linear) epitope prediction

The linear B-cell epitopes of the targeted proteins were predicted by the IEDB (http://tools.iedb.org/bcell/) and the Bepipred 2.0 (http://www.cbs.dtu.dk/services/BepiPred/) server [40]. To predict linear B-cell epitopes, the IEDB employs a combination of sequence features of the antigen, amino acid scales, Emini surface accessibility, and the Hidden Markov model (HMM) approach [41]. Meanwhile, the BepiPred 2.0 server utilizes an HMM and a propensity scale approach, which has a predicted accuracy of 73% [40]. In both instances, the threshold value was set as 0.5. Subsequently, the epitopes were assessed for allergenicity and antigenicity through the AllergenFP v.1.0 (http://ddg-ph armfac.net/AllergenFP/) [42] and VaxiJen 2.0 server (http://www.ddg-ph armfac.net/vaxijen/VaxiJen/VaxiJen.html), respectively [34].

### 2.5 Mapping the vaccine construct

All selected (CTL, HTL, and B-cell) epitopes from S100-A4, S100-A6, S100-A8, S100-A9, and S100-A11 proteins were utilized to construct the vaccine. The selected epitopes were linked together to develop a complete vaccine with recognized adjuvants and suitable linkers. Heparin-binding hemagglutinin (HBHA) (A5TZK3: HBHA_MYCTA) was added as an adjuvant, while five different types of linkers were used to connect the chosen epitopes: EAAAK, AYY, AK, KFER, and GPGPG [43, 44].

### 2.6 Evaluation of physicochemical properties, solubility, allergenicity, and antigenicity

The physicochemical characteristics of the vaccine were assessed following its construction by Expasy’s ProtParam (http://web.expasy.org/protparam/) server. These properties include the total amino acid count, composition and constitute of atoms, molecular weight and formula, total positive and negative residues, stability and aliphatic index, isoelectric point (pI), as well as extinction coefficients and grand average of hydropathicity (GRAVY) [45]. The allergenicity of the vaccine was evaluated by using the AllerTOP v. 2.0 server (https://www.ddg-pharmfac.net/AllerTOP/), which has a prediction accuracy of 85.3% [46]. Alongside, the antigenicity of the vaccine was also assessed by ANTIGENpro (http://scratch.proteomics.ics.uci.edu) (prediction accuracy of 82%) [47] and VaxiJen 2.0 (http://www.ddg-pharmfac.net/vaxijen/ VaxiJen/VaxiJen.html) (prediction accuracy of 70% to 89%) server [34]. In this analysis, the threshold value was set by default setting (0.5). Finally, the solubility of the vaccine was also evaluated by SOLpro (http://scratch.proteomics.ics.uci.edu) (prediction accuracy of 74%) [48–50] and Protein-Sol server (https://protein-sol.manchester.ac.uk/) (prediction accuracy of 58%) [50, 51].

### 2.7 Secondary structure prediction

The PSIPRED (http://bioinf.cs.ucl.ac.uk/psipred/), GOR4 (https://npsa-prabi.ibcp.fr/cgi-bin/npsa_automat.pl?page=/NPSA/npsa_gor4.html) and SOPMA (https://npsa-prabi.ibcp.fr/cgi-bin/npsa_automat.pl?page=/NPSA/npsa_sopma.html) servers were applied to predict and assess the secondary structure of the vaccine [52]. With an accuracy of 84.2%, the PSIPRED predicts the secondary structure of a protein via neural network and PSI-BLAST (position-specific iterated BLAST) [53, 54]. The GOR4 server utilizes both information theory and Bayesian statistics, while the SOPMA uses a neural network to predict the secondary structure with an accuracy of 73.5% [55] and 69.5%, respectively [56]. The FASTA sequence was used to determine the secondary structure in the server mentioned above.

### 2.8 Tertiary structure prediction and validation

The I-TASSER server (https://zhanglab.ccmb.med.umich.edu/I-TASSER/) predicted the vaccine’s tertiary structure (3D). The server utilizes various threading alignments and repeated template segment assembly simulations to determine a protein’s most accurate and precise tertiary structure [29, 44, 57, 58]. The server measures the structure’s confidence score (C-score) when assessing the quality of any predicted 3D model. Since an improved C-score signifies the highest quality or level of confidence of a predicted 3D model. Alongside, the template modeling score (TM-score) and root mean square deviation (RMSD) are typical measures of protein structure similarity, whereas subordinate values provide greater resolution and more accurate 3D model fits [44, 57, 59]. In terms of model prediction accuracy, the I-TASSER models may have an average error of 2 Å for RMSD and 0.08 for TM-score [60]. Consequently, the predicted 3D model of the vaccine was employed for structural refinement through the GalaxyWEB (https://galaxy.seoklab.org/cgi-bin/submit.cgi?type=REFINE) server [61]. Further validation of the model was accomplished by the SAVES v6.0 server (https://saves.mbi.ucla.edu/). The server provides a Ramachandran plot, which defines the stereochemical quality of the predicted vaccine model [62–65]. To identify the structural accuracy of the predicted 3D model structure, we applied the ProSA-web server (https://prosa.services.came.sbg.ac.at/prosa.php). The server provides a Z-score for a predicted 3D model structure, which signifies the accuracy and the potential errors of the model structure [66, 67].

### 2.9 Molecular docking study of the vaccine-TLR receptor

The vaccine must effectively interact with the host’s immunological receptors to elicit a robust immune response. Therefore, protein-protein docking was used to predict the interaction of multi-epitope vaccines with immune receptors, toll-like receptor-2 (TLR-2) and TLR-4. The 3D structure of the vaccine and TLR-2 (PDB ID: 2Z7X) or TLR-4 (PDB ID: 3FXI) were applied to docking using the ClusPro 2.0 server (https://cluspro.bu.edu/login.php), which has a docking accuracy of ~71% [68–72]. However, both TLR-2 and TLR-4 have essential functions in vaccine-induced immunity [73]. These receptors can identify pathogen-associated molecular patterns (PAMPs) and initiate innate and adaptive immune responses. The TLR-2 mainly detects lipoproteins and lipopeptides, while the TLR-4 specifically detects lipopolysaccharides (LPS) [74]. Activating the TLR-2 and TLR-4 by vaccine components triggers a cascade of events that ultimately enhance adaptive immune responses. This includes improved antigen presentation, cytokine generation, and dendritic cell maturation. These processes, in turn, lead to increased antibody synthesis, T-cell activation, and the establishment of immunological memory, all of which significantly improve the effectiveness of vaccination [73, 74]. PyMOL (https://pymol.org/2/) and PDBsum (http://www.ebi.ac.uk/thornton-srv/databases/pdbsum/Generate.html) servers were used to analyze and visualize docked complex structures.

### 2.10 Free energy calculation by molecular mechanics with generalized Born and surface area solvation (MM-GBSA)

The free energy associated with the interaction between the "Vaccine—TLR-2" and "Vaccine—TLR-4" was calculated using MM-GBSA methodologies based on molecular mechanics and the Generalised Born approach. The molecular mechanics approaches under consideration include the influences stemming from bound interactions, van der Waals forces (VDW), electrostatic interactions (ELE), as well as polar (GB) and non-polar (SA) components [75–77]. The polar solvation component is calculated using the Generalised Born equation on the HawkDock server [75–77]. However, the accuracy of the MM-GBSA was reported to be 95.35% and 81.40% for the crystal and predicted structures, respectively [78].

### 2.11 Prediction of B-cell (discontinuous) epitopes

To predict the possible discontinuous B-cell epitopes of the vaccine, we applied the Ellipro of the IEDB database (http://tools.iedb.org/ellipro/) [79]. With the area under the ROC curve (AUC) value of 0.732 and prediction accuracy of 70%, the server utilizes three different algorithms to predict all possible discontinuous B-cell epitopes of the vaccine through their protrusion index (PI) values to illustrate an ellipsoidal protein shape and to quantify the residue PI and neighboring cluster residues [44, 79]. The selection parameters were set to default setting as a minimum score of 0.5 and a maximum distance of 6Å [79].

### 2.12 Codon adaptation and *in silico* cloning

The Java Codon Adaptation tool was employed to perform codon optimization of the vaccine for *in silico* cloning (http://www.jcat.de/) [80]. Therefore, we choose the *Escherichia coli* K12 strain as an expression vector for the vaccine. The codon adaptation index (CAI) value and GC content of the adapted sequence were also collected. Subsequently, the nucleotide sequence adapted to be compatible with the vaccine was introduced into the pET28a(+) vector through the restriction cloning module of the SnapGene software (https://www.snapgene.com/free-trial/). PshAI and Acc65I restriction sites were introduced to ensure suitable insertion into the plasmid.

### 2.13 Immune simulation

To perform the immune stimulation of the vaccine, the C-ImmSim server (https://kraken.iac.rm.cnr.it/C-IMMSIM/) was employed [81]. The server predicts the possible immune response of a mammalian immune system encountered by a vaccine injection. Both the humoral (antibody-mediated) and cellular (cell-mediated) responses were evaluated by this server [80, 82]. For the vaccine, a three-dose vaccination regime with a four-week interval was chosen. Nevertheless, the simulation parameters were configured with the default values, where the number of adjuvants and antigen injections were set to 100 and 1000, respectively [44]. Additionally, the time steps were defined as 1, 84, and 168, where each time step corresponds to 8 hours during daily life. Alongside, the simulation’s volume and steps were adjusted at 50 and 1000, respectively [44]. Without the interference of lipopolysaccharides (LPS), the random seed was set as 12345.

### 2.14 Structural validation of the mRNA vaccine

The secondary structure of the mRNA vaccine was predicted by the RNAfold (http://rna.tbi.univie.ac.at/cgi-bin/RNAWebSuite/RNAfold.cgi) web server [83]. The server can calculate the thermodynamically derived minimum free energy (MFE) of the query mRNA structures with an accuracy of 70% [84–86]. However, the energy parameters were set to default settings: a temperature of 37°C and a 1.021 molar (M) salt concentration. Upon acquiring the optimized DNA sequence via the JCat server, it was then transformed into a possible DNA sequence through the process of DNA<->RNA->Protein conversion at http://biomodel.uah.es/en/lab/cybertory/analysis/trans.htm. This was carried out to facilitate the analysis of mRNA folding and the secondary structure of the vaccine.

## 3. Result

### 3.1 Retrieval of protein sequence

The amino acid sequences of the proteins S100-A4, S100-A6, S100-A8, S100-A9, and S100-A11 were obtained from the NCBI protein database. These retrieved sequences were employed for further analysis.

### 3.2 Cytotoxic T lymphocyte (CTL) epitope prediction

CTL epitopes for five proteins were predicted through the IEDB web server based on their percentile rank (<1.0) and combined score (<1.0). Finally, a total of 73 epitopes were selected following the criteria: percentile rank and combined score <1.0, where 15 epitopes for S100-A4, 13 epitopes for S100-A6, 16 epitopes for protein S100-A8, 11 epitopes for protein S100-A9 and 18 epitopes for protein S100-A11. However, the epitopes were confined to 12 alleles (HLA-A1, HLA-A2, HLA-A3, HLA-A24, HLA-A26, HLA-B7, HLA-B8, HLA-B27, HLA-B39, HLA-B44, HLA-B58 and HLA-B62). The epitopes were assessed for immunogenicity, toxicity, and antigenicity, where they were found to be immunogenic, non-toxic, and antigenic **(Table 1)**.

**Table 1:** The predicted CTL epitope with their immunogenicity, toxicity, and antigenicity.

Protein	Peptides	Combined score	Immunogenicity score	Toxicity	Antigenicity	Alleles
**S100-A4**	STFHKYSGK	0.936641	-0.26275	Non-toxic	Probable antigen	12
	DEAAFQKLM	0.806402	-0.13145	Non-toxic	Probable antigen	12
	NKSELKELL	0.621016	-0.08873	Non-toxic	Probable antigen	12
	RTDEAAFQK	0.286784	0.214	Non-toxic	Probable antigen	12
	VMVSTFHKY	0.295864	-0.10377	Non-toxic	Probable antigen	12
	FLGKRTDEA	0.002737	-0.04184	Non-toxic	Probable antigen	12
	DNEVDFQEY	0.187505	0.16658	Non-toxic	Probable antigen	12
	YSGKEGDKF	0.103906	-0.18388	Non-toxic	Probable antigen	12
	FQKLMSNL	0.121941	0.21434	Non-toxic	Probable antigen	12
	LKELLTREL	0.288341	0.14926	Non-toxic	Probable antigen	12
	FKLNKSEL	0.018919	0.03509	Non-toxic	Probable antigen	12
	ALDVMVSTF	0.169291	-0.20034	Non-toxic	Probable antigen	12
	IAMMCNEFF	0.190912	-0.13939	Non-toxic	Probable antigen	12
	SELKELLTR	0.075372	-0.12022	Non-toxic	Probable antigen	12
	LTRELPSFL	0.121941	0.02509	Non-toxic	Probable antigen	12
**S100-A6**	KELTIGSKL	0.761632	-0.06866	Non-toxic	Probable antigen	12
	GREGDKHTL	0.585583	-0.06482	Non-toxic	Probable antigen	12
	GLLVAIFHK	0.665857	0.31902	Non-toxic	Probable antigen	12
	KHTLSKKEL	0.408604	-0.48616	Non-toxic	Probable antigen	12
	AIFHKYSGR	0.544552	-0.26275	Non-toxic	Probable antigen	12
	LQDAEIARL	0.288341	0.33261	Non-toxic	Probable antigen	12
	NFQEYVTFL	0.236661	0.19957	Non-toxic	Probable antigen	12
	TFLGALALI	0.204377	0.0847	Non-toxic	Probable antigen	12
	ALIYNEALK	0.375627	0.15397	Non-toxic	Probable antigen	12
	CPLDQAIGL	0.196504	0.07487	Non-toxic	Probable antigen	12
	LKELIQKEL	0.130868	-0.0816	Non-toxic	Probable antigen	12
	ARLMEDLDR	0.248374	-0.05832	Non-toxic	Probable antigen	12
	EYVTFLGAL	0.0852	0.20748	Non-toxic	Probable antigen	12
**S100-A8**	SIIDVYHKY	0.977923	0.00354	Non-toxic	Probable antigen	12
	YRDDLKKLL	0.89043	-0.37276	Non-toxic	Probable antigen	12
	KYSLIKGNF	0.844018	-0.11344	Non-toxic	Probable antigen	12
	FHAVYRDDL	0.639914	0.13104	Non-toxic	Probable antigen	12
	LLETECPQY	0.687787	0.04127	Non-toxic	Probable antigen	12
	KMGVAAHKK	0.730005	0.02877	Non-toxic	Probable antigen	12
	SLIKGNFHA	0.672529	-0.02919	Non-toxic	Probable antigen	12
	ALNSIIDVY	0.372198	0.12915	Non-toxic	Probable antigen	12
	IRKKGADVW	0.553195	-0.17433	Non-toxic	Probable antigen	12
	MLTELEKAL	0.459766	0.03766	Non-toxic	Probable antigen	12
	LETECPQYI	0.124868	-0.04951	Non-toxic	Probable antigen	12
	NTDGAVNFQ	0.106585	0.1812	Non-toxic	Probable antigen	12
	SIIDVYHKY	0.977923	0.00354	Non-toxic	Probable antigen	12
	YRDDLKKLL	0.89043	-0.37276	Non-toxic	Probable antigen	12
	KYSLIKGNF	0.844018	-0.11344	Non-toxic	Probable antigen	12
	TIINTFHQY	0.945906	0.14431	Non-toxic	Probable antigen	12
**S100-A9**	RLTWASHEK	0.7835	0.20366	Non-toxic	Probable antigen	12
	VRKDLQNFL	0.768595	-0.10458	Non-toxic	Probable antigen	12
	IMLMARLTW	0.762326	-0.08016	Non-toxic	Probable antigen	12
	VKLGHPDTL	0.466855	0.09296	Non-toxic	Probable antigen	12
	KENKNEKVI	0.377743	-0.28903	Non-toxic	Probable antigen	12
	TFHQYSVKL	0.354928	-0.35655	Non-toxic	Probable antigen	12
	NEKVIEHIM	0.416022	0.30045	Non-toxic	Probable antigen	12
	TNADKQLSF	0.14122	-0.39004	Non-toxic	Probable antigen	12
	LTWASHEKM	0.018919	-0.06088	Non-toxic	Probable antigen	12
	LSFEEFIML	0.015393	0.35617	Non-toxic	Probable antigen	12
	HQYSVKLGH	0.016441	-0.32003	Non-toxic	Probable antigen	12
**S100-A11**	AVFQKYAGK	0.93067	-0.23922	Non-toxic	Probable antigen	12
	GVLDRMMKK	0.945216	-0.37038	Non-toxic	Probable antigen	12
	SLIAVFQKY	0.705995	0.00921	Non-toxic	Probable antigen	12
	NYTLSKTEF	0.765387	-0.2714	Non-toxic	Probable antigen	12
	FLSFMNTEL	0.811116	-0.02173	Non-toxic	Probable antigen	12
	FLKAVPSQK	0.788539	-0.20817	Non-toxic	Probable antigen	12
	GKDGYNYTL	0.627267	0.05117	Non-toxic	Probable antigen	12
	GQLDFSEFL	0.649201	0.12989	Non-toxic	Probable antigen	12
	LSKTEFLSF	0.72244	0.07074	Non-toxic	Probable antigen	12
	YAGKDGYNY	0.494122	-0.1594	Non-toxic	Probable antigen	12
	NLIGGLAMA	0.597566	0.03028	Non-toxic	Probable antigen	12
	CHDSFLKAV	0.300219	-0.21485	Non-toxic	Probable antigen	12
	SPTETERCI	0.218583	0.25758	Non-toxic	Probable antigen	12
	RCIESLIAV	0.189165	0.10759	Non-toxic	Probable antigen	12
	STFHKYSGK	0.936641	-0.26275	Non-toxic	Probable antigen	12
	DEAAFQKLM	0.806402	-0.13145	Non-toxic	Probable antigen	12
	DFSEFLNLI	0.085408	0.13867	Non-toxic	Probable antigen	12
	FMNTELAAF	0.072372	0.1799	Non-toxic	Probable antigen	12

### 3.3 Helper T lymphocyte (HTL) epitope prediction

HTL binding epitopes for the five proteins were predicted through the IEDB web server based on percentile rank <1.0 and screened out that can induce IFN-γ, IL-4, and IL-10 cytokines. Among the 15 selected HTL epitopes, seven were IL-4 non-inducers (non-positive value), and eight were negative to the production of IFN-γ (non-positive value). All of them were found to be IL-10 inducers with positive IL10 scores. Antigenicity, toxicity, and allergenicity were also assessed to select the predicted epitopes for the multi-epitope vaccine construction. We found that 15 epitopes showed antigenicity, non-toxic, and non-allergen activity **(Table 2)**. However, the epitopes were confined to 13 alleles including HLA-DRB1-0101, HLA-DRB1-0301, HLA-DRB1-0401, HLA-DRB1-0701, HLA-DRB1-0801, HLA-DRB1-0901, HLA-DRB1-1001, HLA-DRB1-1101, HLA-DRB1-1201, HLA-DRB1-1301, HLA-DRB1-1401, HLA-DRB1-1501, and HLA-DRB1-1601.

**Table 2:** The predicted HTL epitopes of the selected proteins with their IFN-γ, IL-4, and IL-10 production capability, toxicity, antigenicity, and allergenicity.

Protein	Peptide	IFN-γ	IL-4	IL 10	Antigenicity	Toxicity	Allergenicity	Alleles
**S100-A4**	EAAFQKLMSNLDSNR	0.48896694	-0.01	0.532	Probable antigen	Non-toxic	Non-allergen	13
		Positive	Non-inducer	Inducer				
	GDKFKLNKSELKELL	-0.22472288	1.32	0.632	Probable antigen	Non toxic	Non allergen	13
		Negative	Inducer	Inducer				
**S100-A6**	FQEYVTFLGALALIY	0.31086029	-1.07	0.59	Probable antigen	Non-toxic	Non-allergen	13
		Positive	Non-inducer	Inducer				
	VNFQEYVTFLGALAL	0.1966474	-0.03	0.575	Probable antigen	Non-toxic	Non-allergen	13
		Positive	Non	Inducer				
	VTFLGALALIYNEAL	0.39182418	-0.84	0.513	Probable antigen	Non-toxic	Non-allergen	13
		Positive	Non	Inducer				
**S100-A8**	YHKYSLIKGNFHAVY	0.58980742	0.2	0.618	Probable antigen	Non-toxic	Non-allergen	13
		Positive	Inducer	Inducer				
	DVYHKYSLIKGNFHA	0.60852934	1.23	0.617	Probable antigen	Non-toxic	Non-allergen	13
		Positive	Inducer	Inducer				
	LILVIKMGVAAHKKS	0.51190349	-0.85	0.648	Probable antigen	Non toxic	Non allergen	13
		Positive	Non inducer	Inducer				
**S100-A9**	EEFIMLMARLTWASH	-0.39796618	0.28	0.413	Probable antigen	Non-toxic	Non-allergen	13
		Negative	Inducer	Inducer				
	LSFEEFIMLMARLTW	-0.69921594	-0.70	0.473	Probable antigen	Non toxic	Non allergen	13
		Negative	Non inducer	Inducer				
	QGEFKELVRKDLQNF	-0.12253003	0.20	0.563	Probable antigen	Non toxic	Non allergen	13
		Negative	Inducer	Inducer				
**S100-A11**	KTEFLSFMNTELAAF	-0.067545146	1.27	0.532	Probable antigen	Non-toxic	Non-allergen	13
		Negative	Inducer	Inducer				
	LDFSEFLNLIGGLAM	-0.26248881	-0.76	0.557	Probable antigen	Non-toxic	Non-allergen	13
		Negative	Non-inducer	Inducer				
	SKTEFLSFMNTELAA	-0.20771576	1.34	0.53	Probable antigen	Non toxic	Non allergen	13
		Negative	Inducer	Inducer				
	DGYNYTLSKTEFLSF	-0.50491803	1.42	0.56	Probable antigen	Non-toxic	Non-allergen	13
		Negative	Inducer	Inducer				

### 3.4 B-cell (linear) epitope prediction

A total of 13 linear B-cell epitopes were chosen through the IEDB and the Bepipred 2.0 servers. Two peptide sequences from each protein, three from S100-A4, two from S100-A6, two from S100-A8, three from S100-A9, and three peptide sequences from S100-A11, were selected based on a bepipred score of > 0.5. The peptide sequences were evaluated for their allergenicity and antigenicity using the AllergenFP v.1.0 server and VaxiJen 2.0 server, respectively **(Table 3)**. All of them were found to be probable non-allergens and antigens. However, the peptide sequences’ length varied from 8 to 35.

**Table 3:** The predicted B-cell (linear) epitopes.

Protein	Start	End	Sequence	Length	Allergenicity	Antigenicity
**S100-A4**	21	32	GKEGDKFKLNKS	12	Non-allergen	Probable antigen
	63	71	DSNRDNEVD	9	Non-allergen	Probable antigen
	91	98	EGFPDKQP	8	Non-allergen	Probable antigen
**S100-A6**	21	55	GREGDKHTLSKKELKELIQKELTIGSKLQDAEIAR	35	Non-allergen	Probable antigen
	58	71	EDLDRNKDQEVNFQ	14	Non-allergen	Probable antigen
**S100-A8**	21	37	LIKGNFHAVYRDDLKKL	17	Non-allergen	Probable antigen
	42	67	CPQYIRKKGADVWFKELDINTDGAVN	26	Non-allergen	Probable antigen
**S100-A9**	24	36	VKLGHPDTLNQGE	13	Non-allergen	Probable antigen
	46	57	QNFLKKENKNEK	12	Non-allergen	Probable antigen
	94	111	MHEGDEGPGHHHKPGLGE	18	Non-allergen	Probable antigen
**S100-A11**	27	39	KDGYNYTLSKTEF	13	Non-allergen	Probable antigen
	50	58	FTKNQKDPG	9	Non-allergen	Probable antigen
	68	76	DTNSDGQLD	9	Non-allergen	Probable antigen

### 3.5 Mapping the vaccine construct

**Fig 2:**
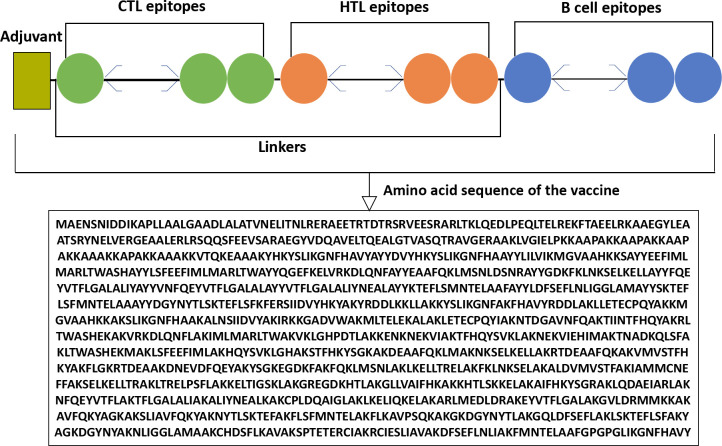
The vaccine construct contains CTL (green color), HTL (orange color), and B-cell epitopes (blue color) with the linkers (adjacent line) and an adjuvant (olive color).

From the selected proteins: S100-A4, S100-A6, S100-A8, S100-A9, and S100-A11, 73 CTL epitopes, 15 HTL epitopes, and 13 B-cell epitopes were chosen for the vaccine construct, which was built by connecting these epitopes with a suitable linker and a precise adjuvant. HBHA was tagged as an adjuvant to the N terminal, while the selected epitopes were linked to each other by using five linkers, including EAAAK, AYY, AK, KFER, and GPGPG **(Fig 2)**.

### 3.6 Evaluation of physicochemical properties, solubility, allergenicity, and antigenicity

The ExPASy ProtPram server illustrated that the vaccine has 1479 amino acids with an MW of 165023.50 Da. The vaccine had an isoelectric point (pI) of 9.45, indicating that it is basic (pH > 7) in nature. The vaccine also had 175 total number of negatively charged and 241 total number of positively charged residues. With the chemical formula of C_7496_H_11807_N_1983_O_2131_S_38,_ and a total number of atoms of 23455, the vaccine also had an extinction coefficient of 147770. However, the estimated high life of the vaccine was found to be different based on the expression system, while it was found to be 30 hours in mammalian reticulocytes, >20 hours in yeast cells, and >10 hours in *E*. *coli*. The server further confirmed the vaccine protein’s stability, which reported an instability index of 23.94 (instability index <40). The aliphatic index of the vaccine was found to be 79.02. Also, the vaccine is expected to be water-soluble (hydrophilic), with a GRAVY score of -0.440. The vaccine is predicted to be firmly soluble upon expression in *E*. *coli* (score of 0.999341). With estimated scores of 0.5283 and 0.4935, the Vaxijen 2.0 and ANTIGENpro servers suggested the vaccine had antigenic properties. The vaccine may not be responsible for any allergic reactions since no allergenicity was predicted in the vaccine through the AllerTOP v. 2.0 server **(Table 4)**. However, the SOLpro and Protein-Sol servers predicted the vaccine as a soluble component with solubility scores of 0.999341 and 0.531, respectively.

**Table 4:** Physicochemical properties, solubility, allergenicity, and antigenicity of the vaccine construct.

Parameter	Value
**Number of amino acids**	1479
**Molecular weight**	165023.50
**Theoretical isoelectric point (pI)**	9.45
**Total number of negatively charged residues (Asp + Glu)**	175
**Total number of positively charged residues (Arg + Lys)**	241
**Formula**	C_7496_H_11807_N_1983_O_2131_S_38_
**Total number of atoms**	23455
**Extinction coefficient (at 280 nm in H2O)**	147770
Estimated half-life (mammalian reticulocytes, *in vitro)*	30 hours
Estimated half-life (yeast cells, *in vivo*)	>20 hours
Estimated half-life (*E*. *coli*, *in vivo*)	>10 hours
**Instability index**	23.94 (Stable)
**Aliphatic index**	79.02
**Grand average of hydropathicity (GRAVY)**	-0.440
**Antigenicity (VaxiJen 2.0)**	0.5283 (Antigen)
**Antigenicity (ANTIGENpro)**	0.4935 (Antigen)
**Allergenicity (AllerTOP v. 2.0)**	Non-allergen
**Solubility (SOLpro)**	0.999341 (Soluble)
**Solubility (Protein-Sol)**	0.531(Soluble)

### 3.7 Secondary structure prediction

**Fig 3:**
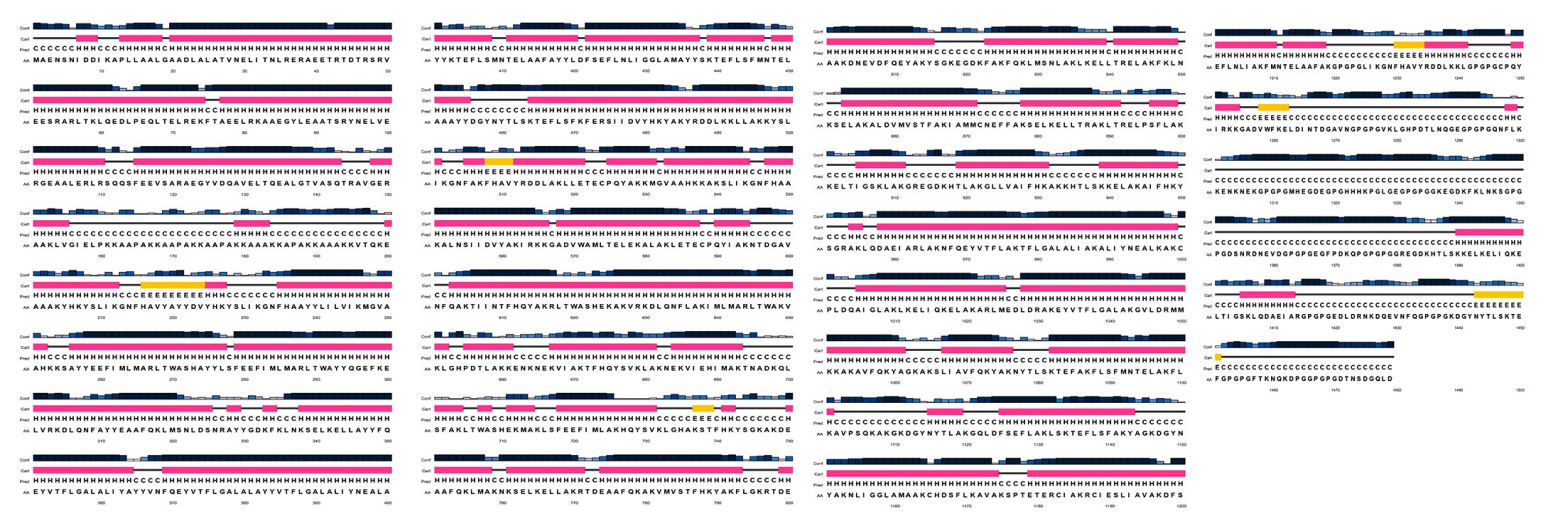
The secondary structure of the vaccine was predicted by the PSIPRED server.

The GOR4, SOPMA, and PSIPRED servers were employed to predict the secondary structure of the vaccine. The GOR4 server demonstrated that the vaccine’s structure comprised 70.18% alpha helix, 25.83% random coil, and 3.99% extended strands (beta sheet). Conversely, the SOPMA server’s prediction for the vaccine’s secondary structure revealed a random coil of 22.38%, an alpha helix of 66.13%, and an extended strand of 6.63%. The SOPMA server predicted a beta-turn structure of 4.87% in the vaccine; however, the GOR4 server did not detect any similar structure **(Table 5)**. Finally, the PSIPRED server provided a three-state prediction for the protein secondary structure, including coil, helix, and strands **(Fig 3, S1 Fig)**.

**Table 5:** The vaccine’s secondary structure properties are predicted using GOR4 and SOPMA servers.

Properties	GOR4	SOPMA
**Alpha helix (Hh)**	70.18% (1038)	66.13% (978)
**310 helix (Gg)**	0.00% (0)	0.00% (0)
**Pi helix (Ii)**	0.00% (0)	0.00% (0)
**Beta bridge (Bb)**	0.00% (0)	0.00% (0)
**Extended strand (Ee)**	3.99% (59)	6.63% (98)
**Beta turn (Tt)**	0.00% (0)	4.87% (72)
**Bend region (Ss)**	0.00% (0)	0.00% (0)
**Random coil (Cc)**	25.83% (382)	22.38% (331)
**Ambiguous states**	0.00% (0)	0.00% (0)
**Other states**	0.00% (0)	0.00% (0)

### 3.8 Tertiary structure prediction and validation

**Fig 4:**
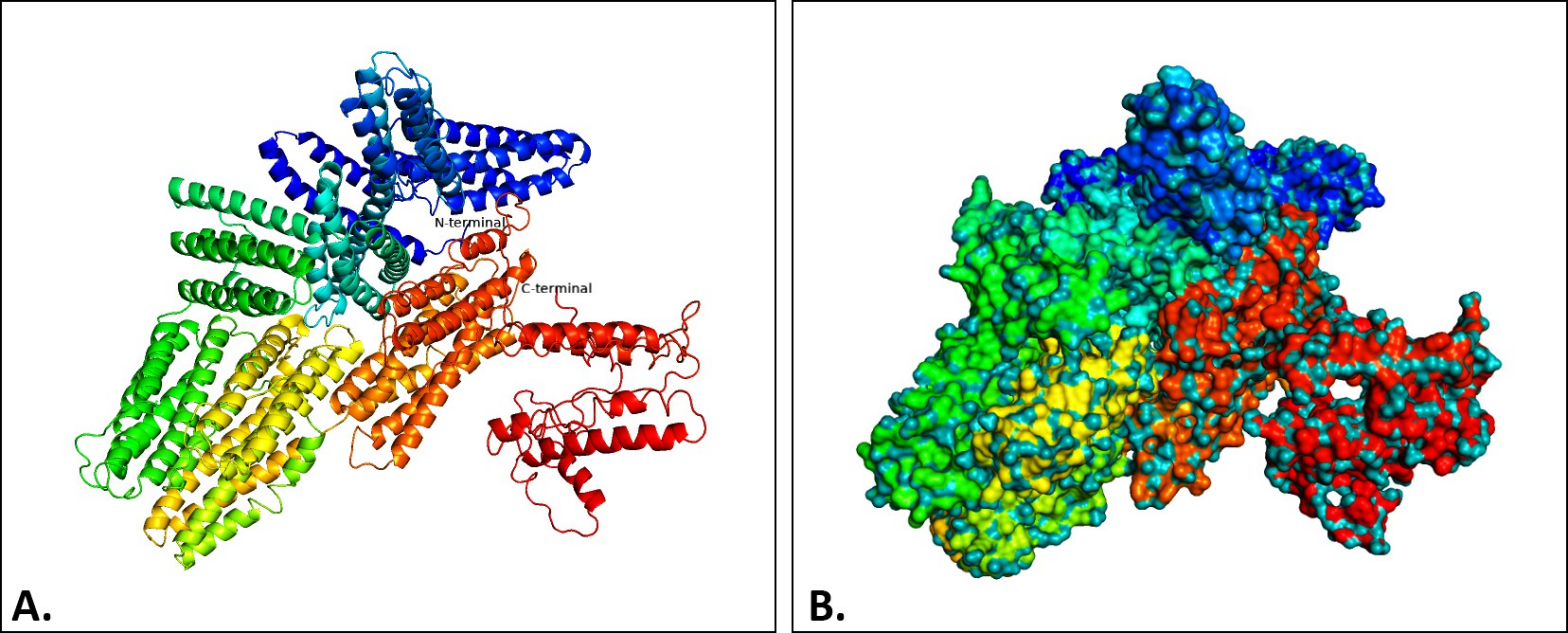
The predicted tertiary structure of the vaccine construct by I-TASSER. The ribbon (A) and surface (B) model view of the vaccine’s tertiary structure was visualized by PyMol software.

**Fig 5:**
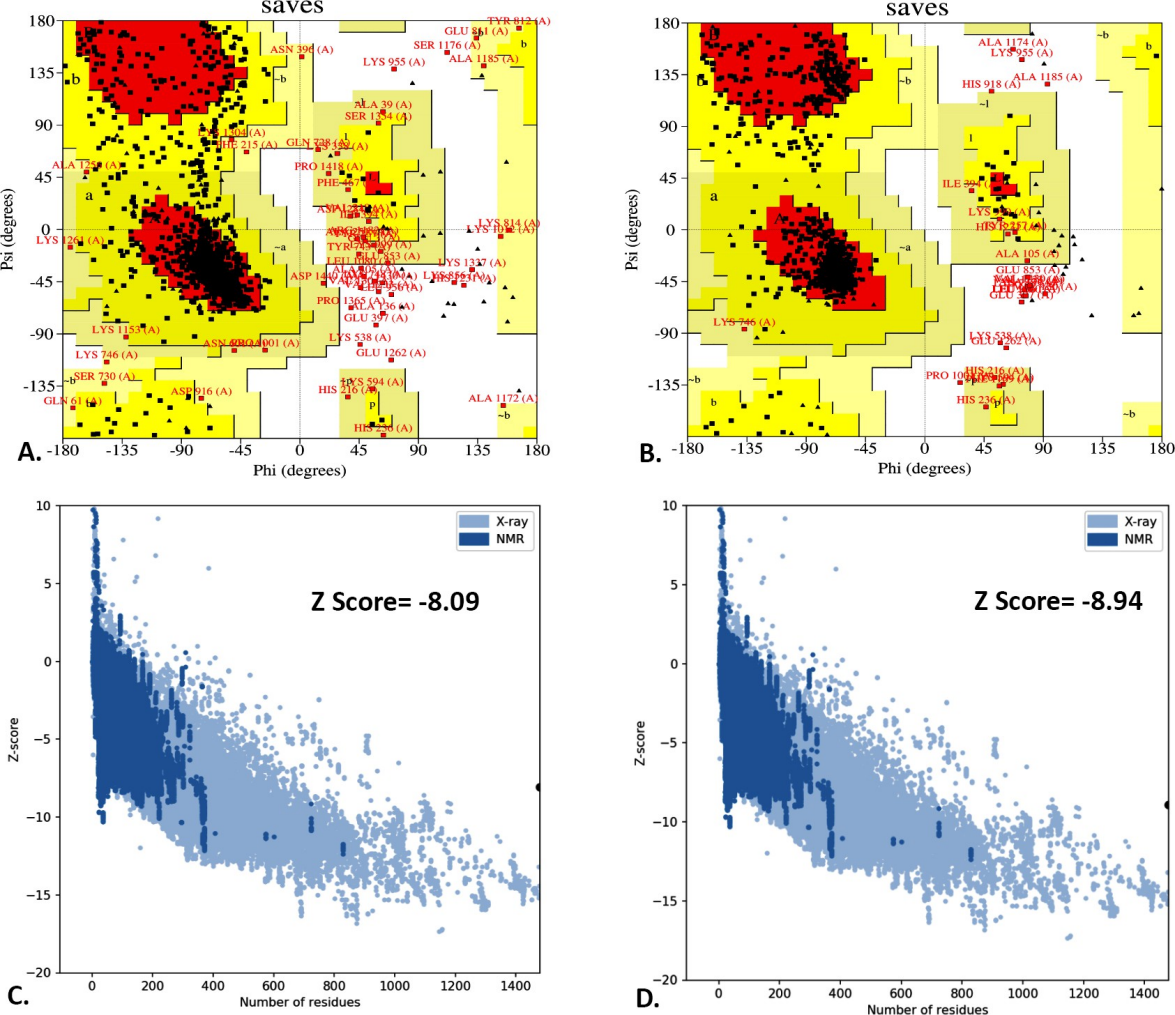
The Ramachandran plot and the Z-score of the predicted tertiary structure before (A and C) and after structural refinement (B and D).

The I-TASSER server predicted five different models for the vaccine structure. Among these, the model was chosen to have the highest C-score of -0.05, TM-score of 0.71 ± 0.12, and RMSD score of 7.1 ± 4.2 Å (**Fig 4**). Subsequently, the refined 3D model was extracted from the GalaxyWEB server, which featured the RMSD, MolProbity score, and Ramachandran’s favorite region values of 0.404, 2.119, and 93.7%, respectively. The Ramachandran plot of the SAVES model demonstrated that the majority of amino acid residues (90.6%) were found in the most favored region, with a smaller percentage (7.5%) in the additional allowed region and a tiny percentage (0.8%) in the generously allowed region **(Fig 5)**. In the unminimized model, the corresponding numbers were 81%, 15.1%, and 2.7%, respectively (**Fig 5, Table 6**). According to the ProSA server, the energy-minimized model exhibited a Z-score of -8.94 **(Fig 5)**, while the unminimized model scored -8.09 (**Fig 5**). Additionally, the SWISS-MODEL predicts that the vaccine had a MolProbity score of 2.93, a Ramachandran preferred area of 81.31%, a QMEAN score of -7.88, and a QMEANDisCo Global score of 0.27± 0.05 before refinement, while after refinement these values were found to be 1.94, 94.11%, -3.88 and 0.29 ± 0.05, respectively **(Table 6)**.

**Table 6:** The quality assessment and structural validation of predicted tertiary structure.

Model	SAVES		ProSA	SWISS-MODEL Structure Assessment			
	PROCHECK (Ramachandran favored region)	ERRAT	Z score	MolProbity Score	Ramachandran favored region	QMEAN	QMEANDisCo Global
**I-Tasser (after refinement)**	90.6%	90.77	-8.94	1.94	94.11%	-3.88	0.29 ± 0.05
**I-Tasser (before refinement)**	81%	91.33	-8.09	2.93	81.31%	-7.88	0.27± 0.05

### 3.9 Molecular docking between the vaccine and TLR receptor

**Fig 6:**
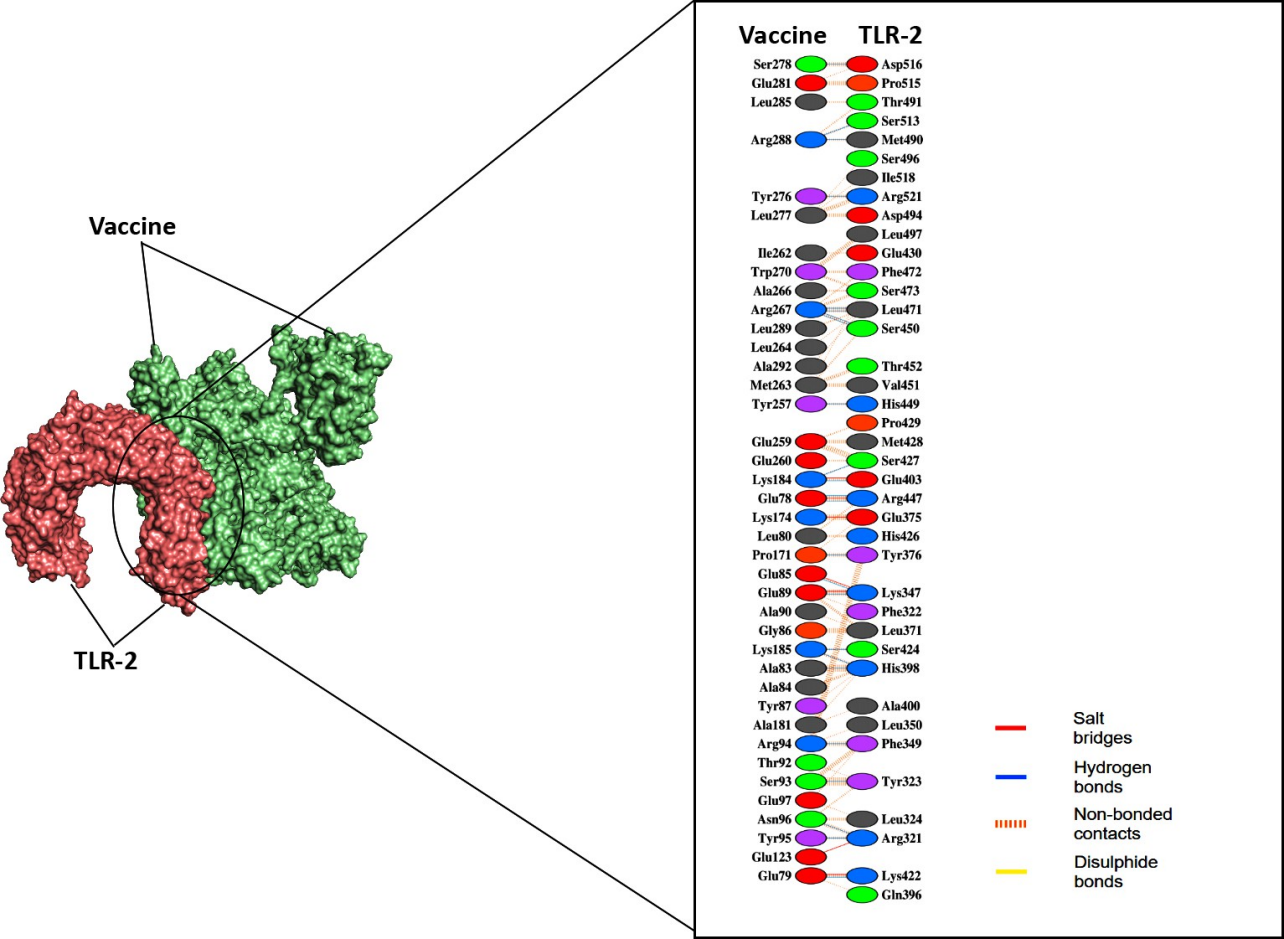
The docked complex of “Vaccine—TLR-2” and their interacting amino acid residues predicted by the Cluspro 2.0 server.

**Fig 7:**
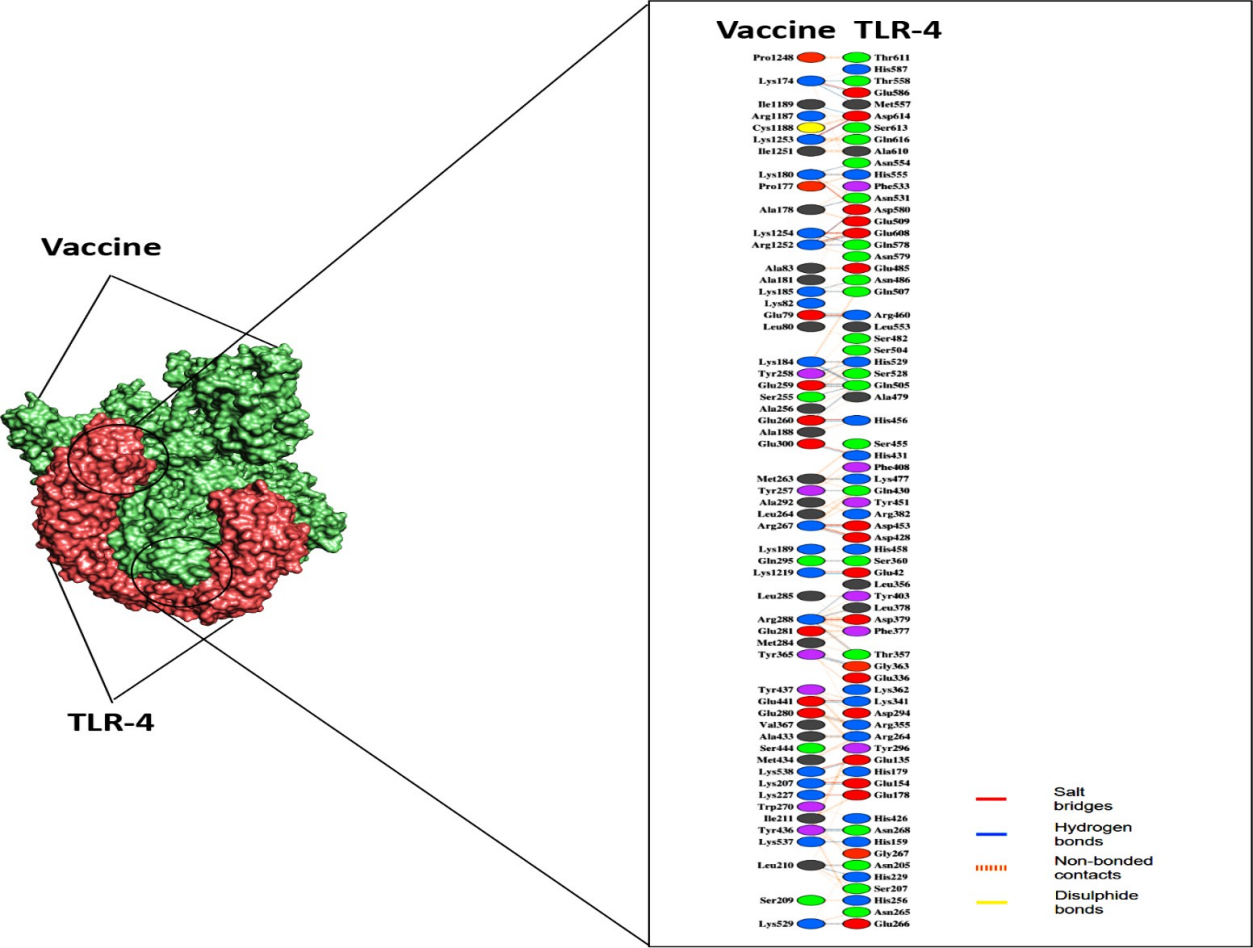
The docked complex of “Vaccine—TLR-4” and their interacting amino acid residues predicted by the Cluspro 2.0 server.

The ClusPro 2.0 server was used to perform molecular docking and confirm the possible interactions of the construct with the TLR-2 and TLR-4 receptors. ClusPro 2.0 generated 60 docked structures for each receptor. Among these generated models, the preferred ones were chosen based on the highest binding affinity and the lowest intermolecular energy. When docking with TLR-2 and TLR-4, the predicted lowest energy scores were -1031.7 (kJ/mol) and -1313.6 (kJ/mol), respectively. Subsequently, PyMOL and PDB-sum were used to analyze and visualize the docked vaccine-TLRs complex structures, and based on the details provided by PDBsum, the "Vaccine—TLR-2" complex had seven hydrogen bonds, 24 salt bridges, and 251 non-bond interactions **(Fig 6, S1 Table)**. Besides, the "Vaccine—TLR-4" complex contained 48 hydrogen bonds, 17 salt bridges, and 446 non-bond interactions **(Fig 7, S1 Table)**.

### 3.10 Free energy calculation by MM-GBSA

We calculated the free binding energy (MM-GBSA) for the vaccine-receptors complexes through the HawkDock server. For the “Vaccine—TLR-2” complex, the VDW, ELE, GB, and SA were calculated to be -216.29 (kcal/mol), -1451.89 (kcal/mol), 1556.13 (kcal/mol), and -29.02 (kcal/mol), respectively. Subsequently, a total binding free energy of -141.07 (kcal/mol) was calculated for the complex. Regarding the “Vaccine—TLR-4” complex, the VDW, ELE, GB, and SA were estimated to be -356.85 (kcal/mol), -8958.65 (kcal/mol), 9092.05 (kcal/mol), and -48.28 (kcal/mol), respectively. However, an elevated binding free energy was calculated for the complex, estimating -271.72 (kcal/mol) **(S2 Fig)**.

### 3.11 Prediction of B-cell (discontinuous) epitopes

**Fig 8:**
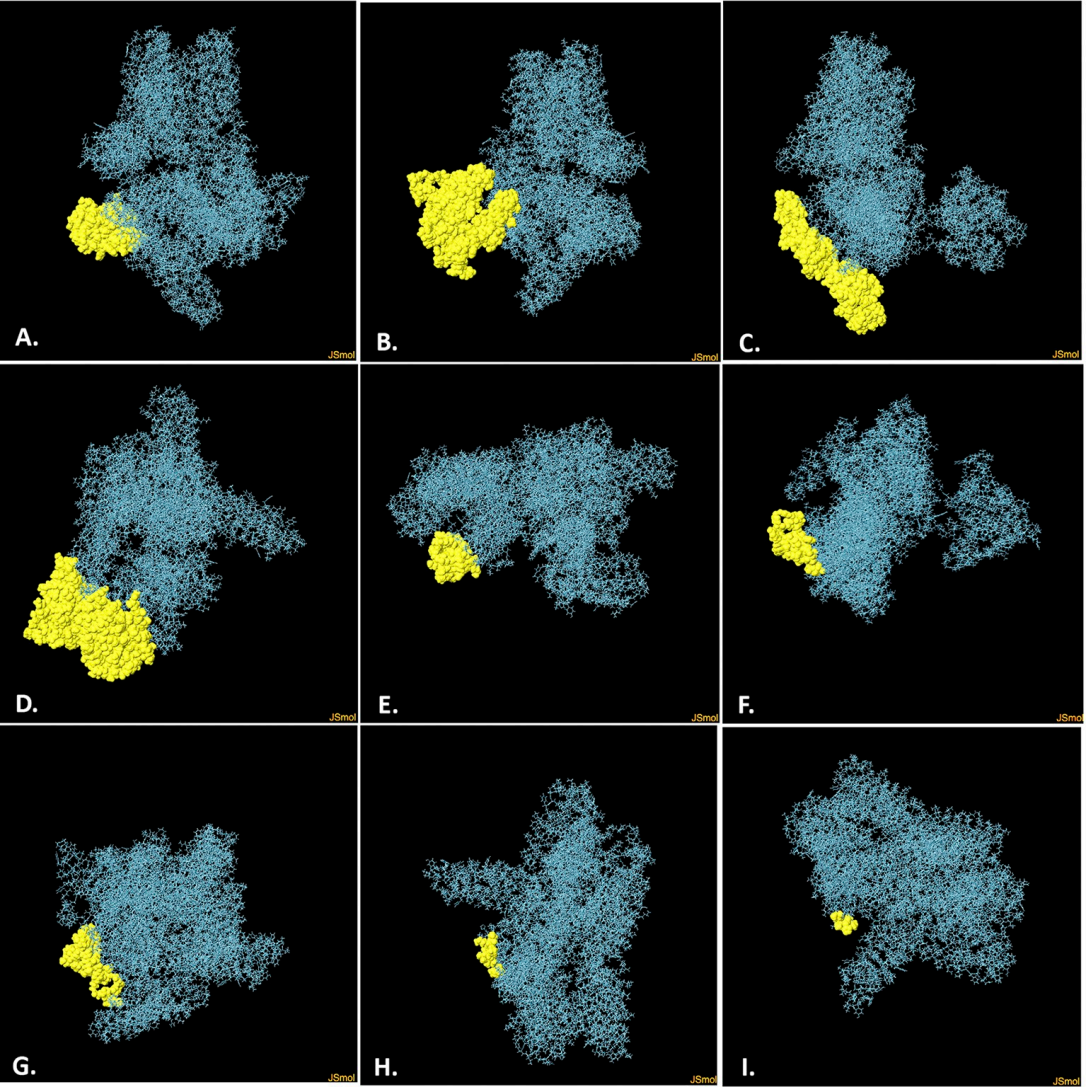
The predicted discontinuous B-cell epitopes of the vaccine (A–I). Yellow surfaces indicate the predicted discontinuous B-cell epitopes, while cyan sticks reveal the vaccine.

With a total of 746 amino acid residues, the Ellipro server identified nine discontinuous B-cell epitopes in the vaccine (**S1 Table**). However, each of these epitopes has a number of residues and a score range ranging from 0.535 to 0.792. (**Fig 8, S2 Table**).

### 3.12 Codon adaptation and *in silico* cloning

**Fig 9:**
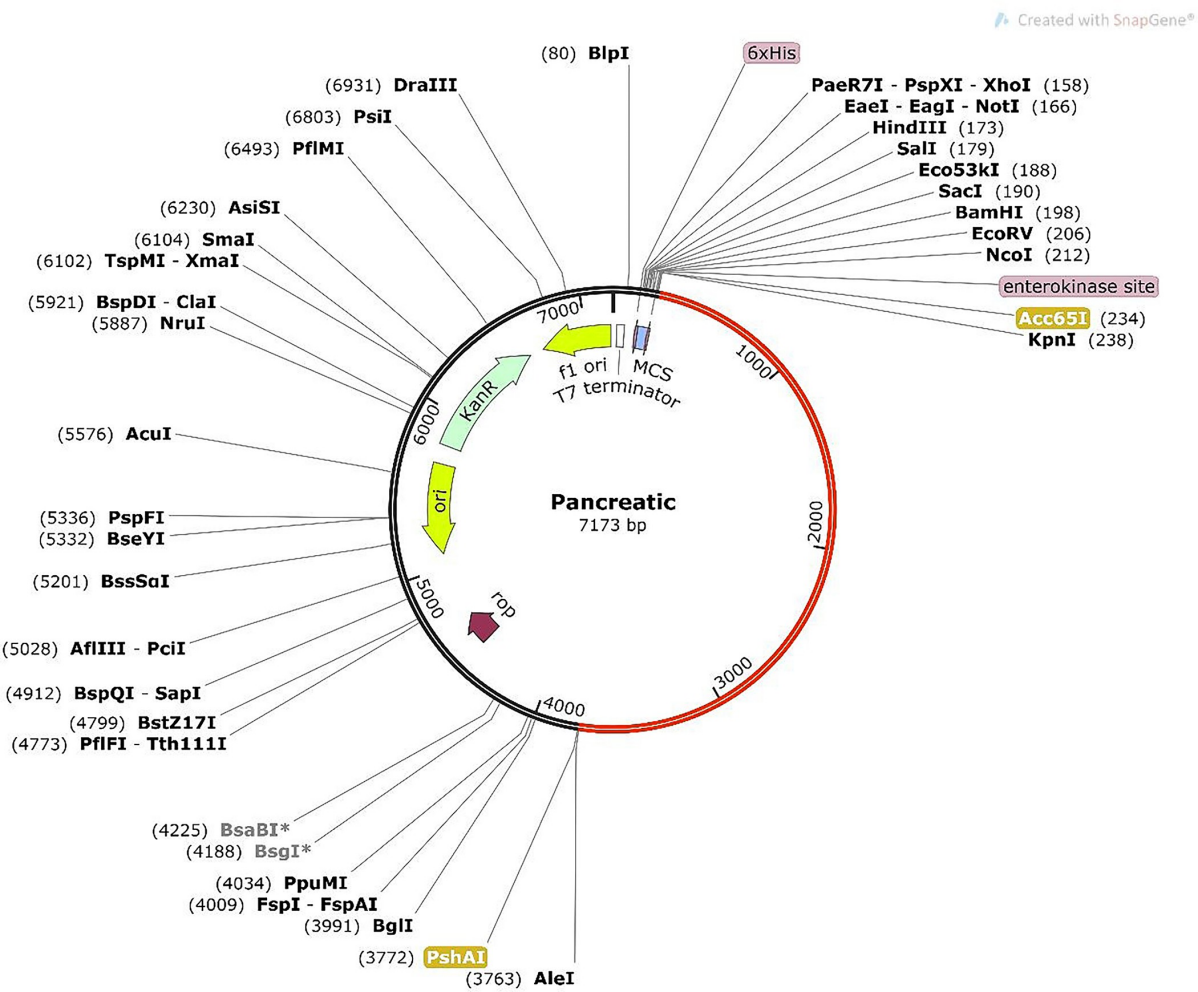
*In silico* cloning of the vaccine’s optimized codon sequences into pET28a (+) vector. Restriction sites indicated in yellow boxes show the two restriction sites (Acc65I and PshAI).

*In silico* cloning was performed primarily to introduce the vaccine into the *E*. *coli* expression system. Following that, we adapted the codons of the vaccine in the *E*. *coli* K12 expression system through the JCAT server. In this analysis, we found that the GC content of the improved sequence was 47.4% while the CAI value was 1.0, which was satisfactory. The CAI is an approach for assessing the biases in synonymous codons for a certain target sequence. However, the CAI index’s value may vary from 0 to 1, suggesting the possibility of a successful expression. The higher the score, the more likely the target gene will be expressed. Meanwhile, the GC content is between 30 and 70%, corresponding to the optimal range. Finally, the optimized codon sequence was inserted in the pET28a (+) vector between PshAI and Acc65I restriction sites by Vector NTI Advance software **(Fig 9)**.

### 3.13 Immune simulation

**Fig 10:**
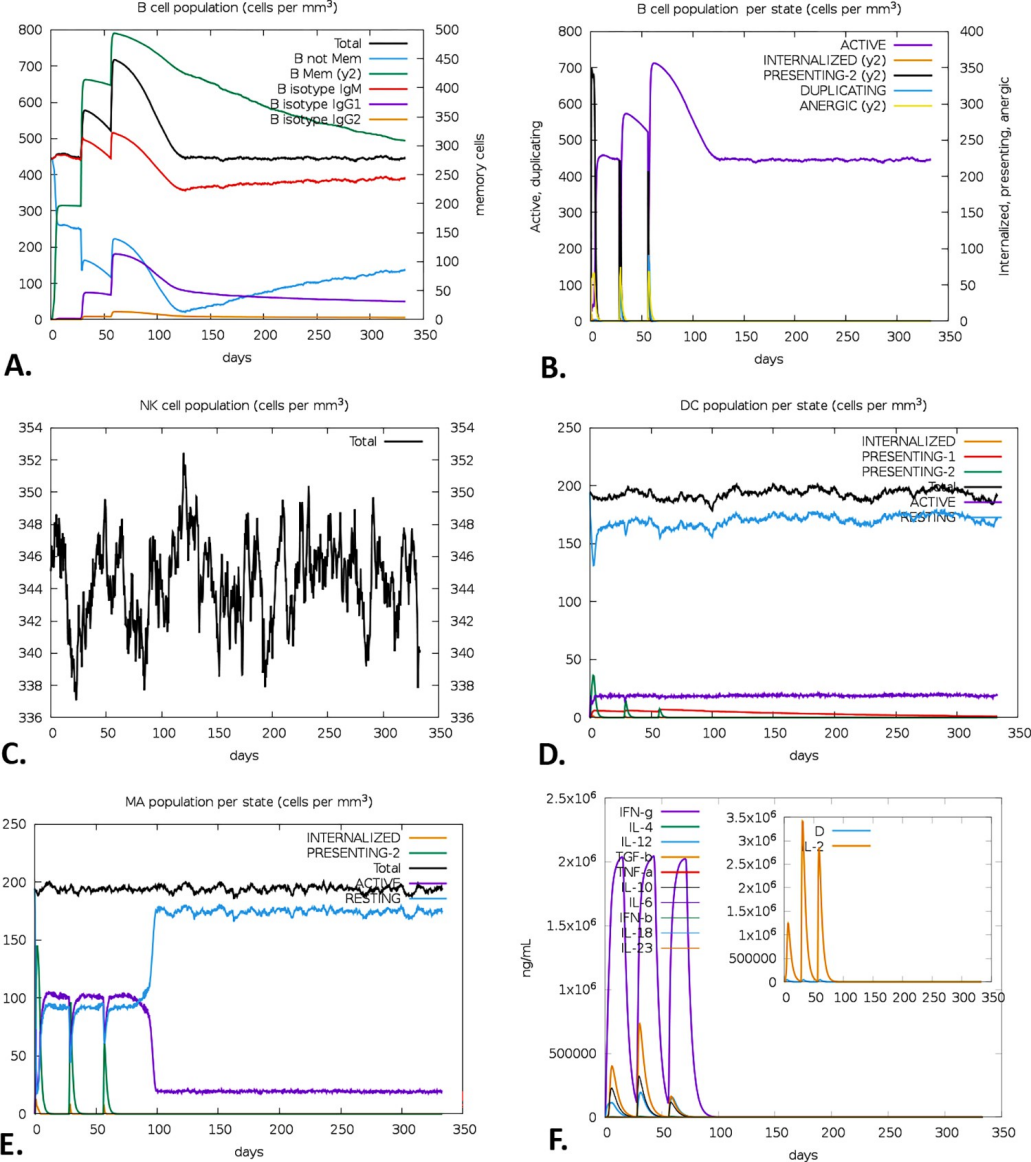
Exploring the vaccine’s immune simulation using the C-ImmSim server. The evolution of entire (A) and per state (B) B-cell populations, NK (C) and DC (D) cell populations, the population of Mφ per state (E), and the cytokines and the IL-2 level are illustrated by the primary plot and the sub-plot, respectively (D) (Here, D refers to Simson’s index, which measures the degree of variety. Since an increase in D suggests an increase in the number of epitope-specific T-cells, a lower D value indicates a lower level of diversity).

**Fig 11:**
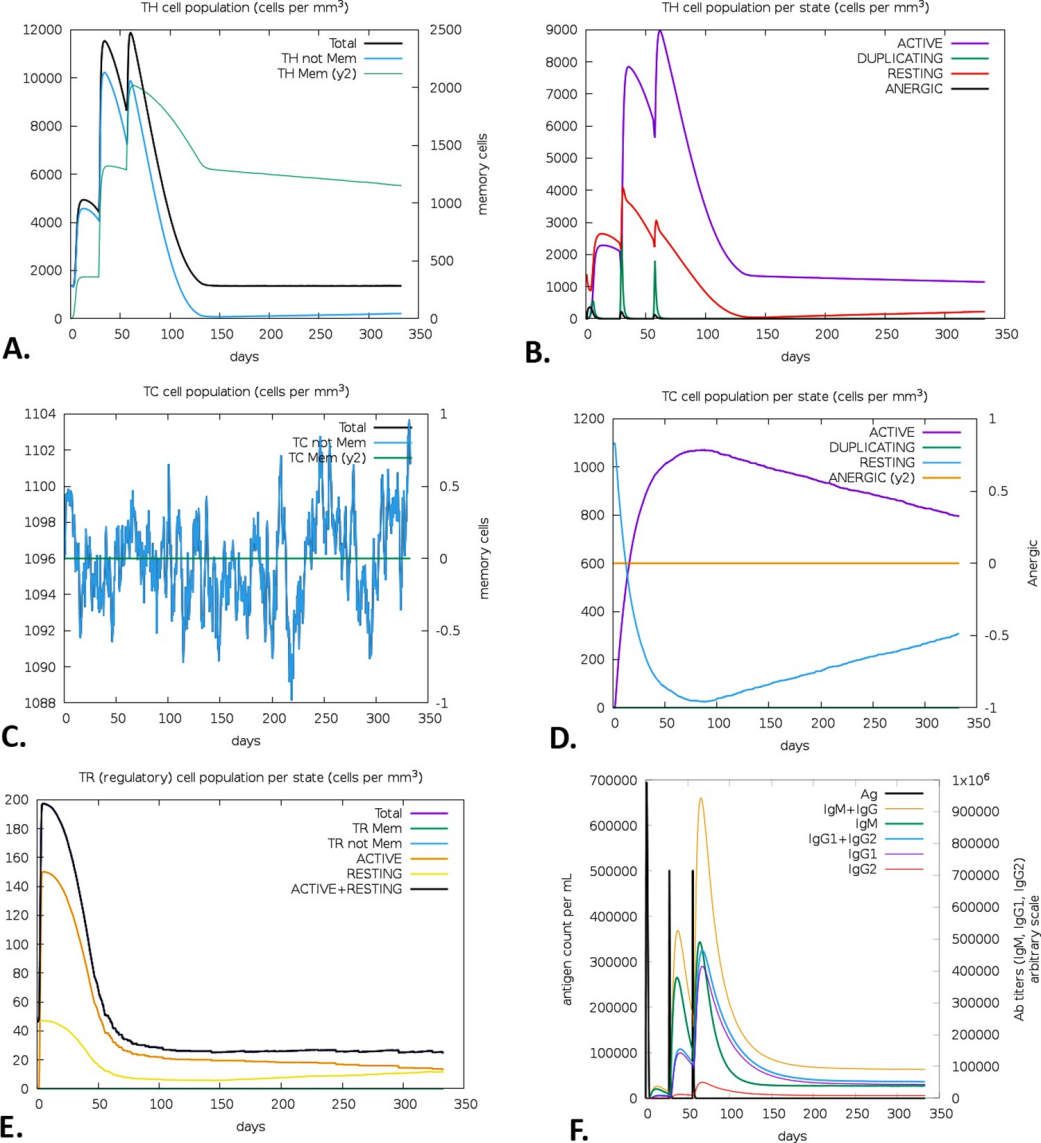
T-cells mediated immune responses predicted by the C-ImmSim server. The evolution of Th with their memory cell life span (A), Th cell population per state cell (B), the development of entire Tc populations (C) and Tc population per state cell (D), the Treg populations per state (E), and the antigen and antibody titers after post vaccination state (F).

Within 60 days of receiving the immunization, a high expression of B-cells became apparent, along with an increased memory B-cell count (**Fig 10A**). Every B-cell was functioning, and the immune response persisted for almost a year (**Fig 10B**). The natural killer (NK) and dendritic cell (DC) cells also showed substantially long-lasting immunity **(Fig 10C and 10D)**. After vaccination, the total macrophage (Mφ) population remained constant for a year **(Fig 10E)**. Furthermore, the vaccination resulted in a substantial rise in the production of IFN-γ while concurrently suppressing the expression of tumor growth factor-β (TGF-β), conferring a robust immune response that persisted for two months (**Fig 10F**). Following vaccination, the observed T-cell responses include Th cells, CTL cells, and regulatory T-cells (Treg). The research further demonstrated a high expression level of both active Th cells and memory Th cells on day 60 following immunization, but that level declined with time (**Fig 11A and 11B**). Additionally, functional CTLs were identified as a high-level expression that remains relatively long (**Fig 11C and 11D**), whereas Treg cells reduced significantly after the immunization (**Fig 11E**). The total amount of antigens was observed for 50 days of vaccination, which was further replaced by IgM+IgG **(Fig 11F)**.

### 3.14 Structural validation of the mRNA vaccine

**Fig 12:**
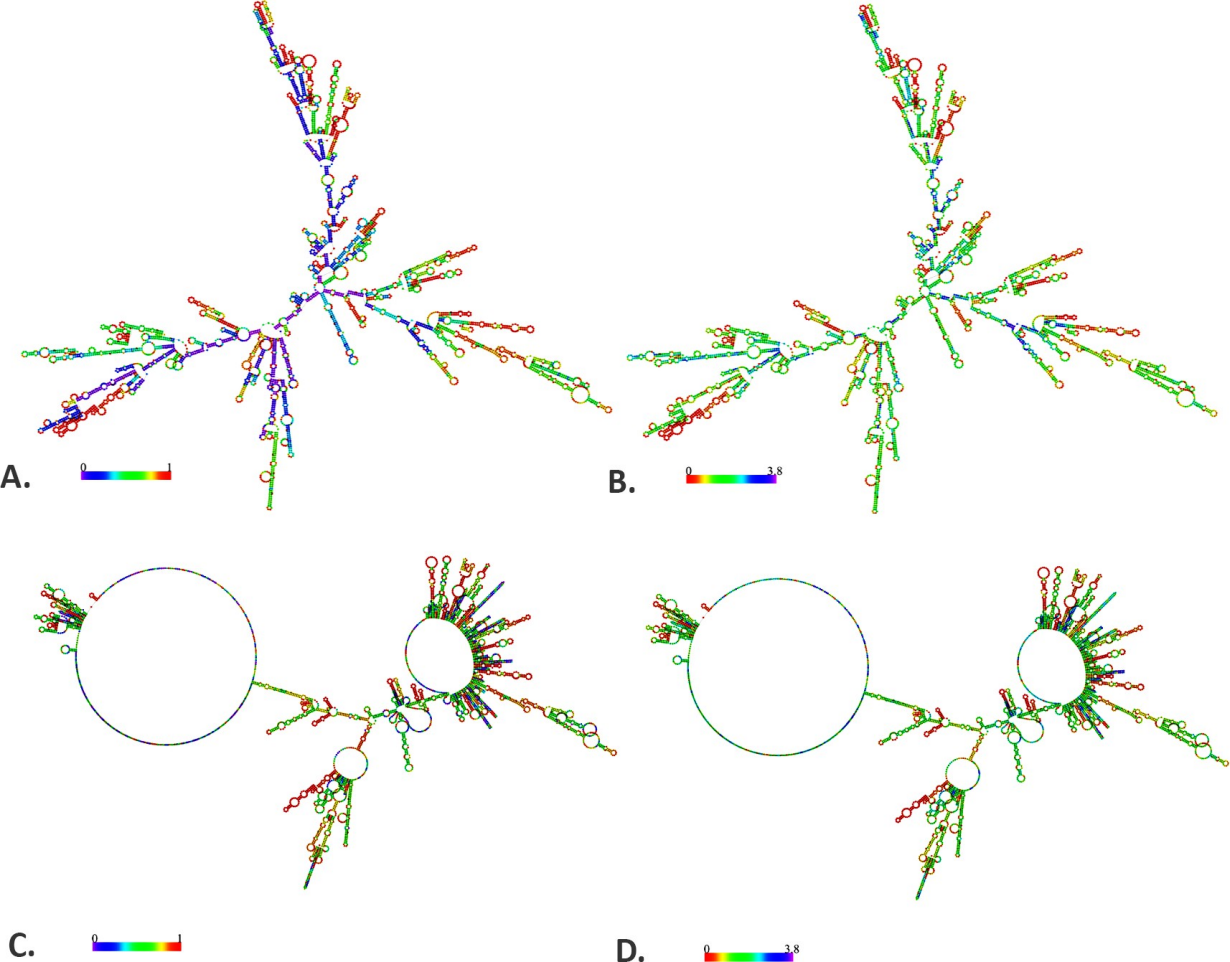
Predicted mRNA structure of the vaccine by RNAfold web server. The base pair probabilities of the mRNA vaccine with the minimum free energy (A) and centroid (B) structure and the positional entropy of the mRNA vaccine with the minimum free energy (C) and centroid (D) structure.

The secondary structure of the vaccine mRNA sequence was illustrated by the RNAfold server with an MFE score of -1760.00 kcal/mol (optimal secondary structure) and -1211.70 kcal/mol (centroid secondary structure). The free energy of the thermodynamic and the frequency of the MFE structure in the ensemble were predicted at -1818.19 kcal/mol and 0.00%, respectively. The prediction of the secondary structure of the mRNA vaccine is depicted in Fig 12 and S3 Fig. This result is consistent with previous research suggesting that the mRNA structure of the current vaccine may remain stable following its entry, transcription, and expression in the host [85, 87–90].

## 4. Discussion

In a notable scientific advancement, researchers successfully developed the first-ever cancer vaccine in the year 1980. This groundbreaking vaccine was created using tumor cells and tumor lysate, especially autologous tumor cells, in the development of colorectal cancer treatment [91, 92]. In the early 1990s, the discovery of the first human tumor antigen, melanoma-associated antigen 1 (MAGE-1), paved the way for further exploration and utilization of tumor antigens in developing potential cancer treatments [93]. Cancer vaccines primarily use tumor-associated antigens (TAAs) and tumor-specific antigens (TSAs) to stimulate the individual’s immune system. In principle, the vaccine’s administration can elicit targeted cellular immunity and humoral immune response, impeding the progression of tumors and eventually eradicating malignant cells [94]. In the meantime, most cancer vaccines are undergoing preclinical and clinical trials [95]. Therefore, there is an urgency to develop more precise antigens and platforms for cancer vaccine development.

Over the last decade, significant advances in technology and research investment have shown that the fast-expanding area of mRNA therapeutic agents has become a viable platform for addressing many challenges encountered in vaccine development for infectious diseases and cancer [96, 97]. Since mRNA is a non-infectious and non-integrating platform, there are no possible hazards of infection or insertional mutagenesis, thus making it an advantageous vaccine candidate over subunit, killed, live-attenuated, and DNA-based vaccines [98–101]. Furthermore, the in vivo stability of mRNA may be modulated by several changes and delivery approaches since it undergoes degradation via intrinsic cellular mechanisms [98–101]. Additionally, there is also a prospect of modifying and downmodulating the immunogenicity of mRNA vaccines to improve their safety aspect [102]. mRNA vaccines may also have additional benefits, such as low production costs, rapid development, and vaccine effectiveness. Since they can be expressed in the cytoplasm without reaching into the nucleus, they perform better than DNA vaccines [103].

The manipulation of mRNA sequences has the potential to enable the manufacture of a wide range of targeted proteins with novel therapeutic applications. Computational approaches have emerged as more efficient for recognizing vaccine compositions than conventional vaccine development techniques. These approaches leverage the power of advanced algorithms and data analysis techniques to expedite the process of identifying optimal vaccine compositions. By analyzing vast amounts of data, these methods can quickly identify potential vaccine candidates and predict their efficacy [104]. Utilizing existing manufacturing processes offers advantages like enhanced diversity, flexibility, time efficiency, and cost-effectiveness in protein production [101, 105–107]. Despite being aware of some intrinsic constraints such as immunogenicity, instability, and delivery inefficiency, mRNA vaccines have shown promising signs owing to recent advancements in synthesis technology and structural alterations of mRNA sequences [108–110]. Recently, the FDA granted authorization over the first two SARS-CoV-2 mRNA vaccines, particularly Pfizer/BioNTech’s BNT162b2 and Moderna’s mRNA-127 (Spikevax) [111, 112]. Also, Pfizer/BioNTech’s BNT162b2 is the first mRNA vaccine to get commercial approval from the FDA [111, 112]. In 2023, Professors Katalin Kariko and Drew Weissman received a "Nobel prize" in physiology or medicine for the innovations of such SARS-CoV-2 mRNA vaccines [113]. The advent of these innovative vaccines has ushered in an age of innovation in the field of vaccination against infectious diseases and cancer.

The mRNA vaccine platform shows promise as a potential strategy for cancer vaccines since it involves the introduction of exogenous synthesized mRNA into cells to serve as templates for antigen production [97, 114]. Non-replicating mRNA and self-amplifying RNA vaccines are the two primary categories of mRNA vaccines. However, most mRNA-based cancer vaccines have been formulated by non-replicating mRNA [97, 103]. Moreover, mRNA vaccines enable the concurrent encoding of multiple antigens, including full-length tumor antigens. The elicitation of increased humoral and cellular immune responses by the encoded antigens enhances the potential to overcome resistance to cancer vaccines [115]. Also, the mRNA vaccine has shown promising results in stimulating MHC I-mediated CD8+ T-cell responses, which makes it a potential candidate for cancer treatment [116, 117]. Recently, Pfizer and BioNTech developed an mRNA neoantigen vaccine against PDAC. The vaccine is based on uridine mRNA-lipoplex nanoparticles, demonstrating a substantial efficacy level in the phase I clinical trial [118]. However, this study is the first endeavor to develop an *in silico*-based mRNA vaccine for pancreatic cancer. Therefore, this study involves the identification of various overexpressed protein members of the S100 protein family, including S100-A4, S100-A6, S100-A8, S100-A9, and S100-A11, to develop a successful multiepitope-based vaccine against pancreatic cancer. CTL epitopes are vital in stimulating the host immune responses to combat intracellular pathogens. The activation of Tc cell response originates through the binding of Tc cells to MHC-I molecules. Hence, vaccines formulated using CTL epitopes can induce robust CD8+ T cell activation, thus contributing a significant role in eradicating intracellular pathogens [119]. Furthermore, HTL epitopes are crucial in presenting immunogenic processed peptides to the T-cell receptor (TCR) on CD4+ T-cells. Therefore, it is pivotal in initiating both cellular and antibody-mediated immune responses. The association between MHC-II molecules and the TCR plays a key role in defense against microbial infections, rejecting transplants, and tracking the progression of malignancies [58, 120–123]. Consequently, the development of mRNA vaccines always requires recognition by CD4+ and CD8+ T-cells [80, 124, 125]. This study evaluated the epitopes from S100-A4, S100-A6, S100-A8, S100-A9, and S100-A11 for their ability to bind to MHC-I and MHC-II on immune cells. Regarding CTL epitopes, the presentation of peptides tends to be restricted to specific alleles, including HLA-A1, HLA-A2, HLA-A3, HLA-A24, HLA-A26, HLA-B7, HLA-B8, HLA-B27, HLA-B39, HLA-B44, HLA-B58 and HLA-B62. The HTL-predicted peptides were restricted to specific alleles, including MHC-II alleles such as HLA-DRB1-0101, HLA-DRB1-0301, HLA-DRB1-0401, HLA-DRB1-0701, HLA-DRB1-0801, HLA-DRB1-0901, HLA-DRB1-1001, HLA-DRB1-1101, HLA-DRB1-1201, HLA-DRB1-1301, HLA-DRB1-1401, HLA-DRB1-1501, and HLA-DRB1-1601. The peptides demonstrated an elevated level of antigenicity while showing minimal levels of allergenicity and toxicity.

B-cell epitopes have been extensively recognized as a fundamental aspect in the development of vaccines since they play a substantial role in the association between antigens and antibodies [80, 126–128]. The B-cell epitopes we anticipated exhibited an elevated antigenicity level and lacked any allergenicity indications. Subsequently, various linkers and adjuvants were used to fabricate the vaccine. Additionally, the designed mRNA vaccine was estimated to have an MW of 165023.50 Da and was found to be highly soluble. Assessing the solubility of a recombinant protein in overexpressed *E*. *coli* is of utmost importance for a diverse range of biochemical and functional experiments [80, 129]. The vaccine’s theoretical pI is 9.45, suggesting acidic characteristics. It also has instability and aliphatic indexes of 23.94 and 79.02, respectively. These values indicate that the vaccine exhibited hydrophobic characteristics consistent with the reported presence of aliphatic side chains. Vaccine development relies heavily on understanding protein folding into secondary and tertiary structures [80, 130]. Structural antigens, such as those in regions of unfolded protein and α-helical coils, have been crucial for eliciting protein-specific immune responses. Antibodies formed in response to opportunistic infections can bind to these two structural antigens if they refold into their native shape. Refining the vaccine led to a notable improvement in its tertiary structure, unveiling key features on the Ramachandran plot. The Ramachandran plot analysis revealed that a substantial majority (90.6%) of the vaccine residues are in preferred areas. Besides, a significant portion of the residues (7.05%) are located in the allowed regions, with a smaller fraction (0.8%) found in the generously allowed region. Based on the findings, the vaccine model’s overall quality is satisfactory. A docking study using human TLR-2 and TLR-4 evaluated the interaction between the vaccine and TLRs on immune cells. Subsequently, the docking analysis suggested that the vaccine had a significant level of affinity for TLR-2 and TLR-4 receptors. The MM-GBSA analysis of the "Vaccine—TLR-2" and "Vaccine—TLR-4" complexes also suggest a strong binding affinity with a free binding energy score of -141.07 (kcal/mol) and -271.72 (kcal/mol), respectively.

Codon optimization was performed to improve the expression of our recombinant vaccine in *E*. *coli*, especially the K12 strain, which provides a high degree of expression of the vaccine in bacteria with a GC content of 47.04% and CAI score of 1.0 (acceptable range for GC content is 30–70%, and for CAI is 1.0). The appearance of memory B-cells and T-cells was also observed, along with the persistent immunity of B-cells over one year. The activation of Th and subsequent production of IFN-γ and IL-2 exhibited distinctive features, evidenced by the immediate rise in IFN-γ and IL-2 concentrations after the first administration and their persistent elevation at maximum levels with repeated exposure to the antigen. This finding suggests an increase in the levels of Th cells and the production of IgM and IgG, which indicates a humoral immune response. Also, the minimum free energy of the mRNA vaccine was predicted to be -1760.00 kcal/mol, indicating the stability of the vaccine following its entry, transcription, and expression in the host [85, 87–90, 131].

## 5. Conclusion

*In silico*-based mRNA pancreatic cancer vaccines represent a promising and innovative approach to cancer immunotherapy. The vaccines are designed using computational methods to identify and encode tumor-specific antigens into mRNA molecules, which can be delivered to the patient’s immune cells to stimulate a robust anti-cancer immune response. The developed mRNA vaccine appeared to be soluble, hydrophilic, and acidic. The structural analysis revealed that the vaccine was a stable and functioning protein. Following that, the docking study indicated that the vaccine has a high affinity for TLR-2 and TLR-4 receptors, whereas the MM-GBSA analysis validated the statement. The vaccine was also firmly expressed in a computationally designed bacterial vector. Regarding immunological responses, the vaccine showed both humoral and adaptive immunity. Finally, this mRNA vaccine would be stable enough after its entrance, transcription, and expression in the host. The findings from these studies provide valuable insights into the properties and potential applications of a successful computationally designed PDAC vaccine. The development of this vaccine marks a significant milestone in the field of PDAC research and therapeutic advancements.

## Supporting information

S1 Fig The secondary structure of the vaccine was predicted by the PSIPRED server, with the features (A) and types (B) of amino acids of the vaccine. In the sequence, among 1479 amino acids, where most of the amino acids were in the coil structure (grey), lesser in the helix structure (pink color), and least in the strand (yellow color) (A). The different types of amino acids in the sequence have been exhibited: the small nonpolar amino acids were predominant (orange), the hydrophobic amino acids (green) and the polar amino acids (red) were less prominent, and the aromatic plus cysteine residues (sky blue) were least prominent (B).
(TIF)

S2 Fig The MM-GBSA free binding energy analysis of the “Vaccine—TLR-2" and "Vaccine—TLR-4" complexes. (TIF)

S3 Fig A mountain plot representation of the MFE structure, the thermodynamic ensemble of RNA structures, and the centroid structure. (TIF)

S1 Table The interactions between the vaccine and TLRs. (DOCX)

S2 Table The discontinuous B-cell epitopes of the vaccine by Ellipro server. (DOCX)

## References

[1] Adamska A, Domenichini A, Falasca M. Pancreatic Ductal Adenocarcinoma: Current and Evolving Therapies. Int J Mol Sci. 2017;18(7):1338. doi: 10.3390/ijms18071338 28640192 PMC5535831

[2] Rahib L, Smith BD, Aizenberg R, Rosenzweig AB, Fleshman JM, Matrisian LM. Projecting cancer incidence and deaths to 2030: the unexpected burden of thyroid, liver, and pancreas cancers in the United States. Cancer Res. 2014;74(11):2913–21. doi: 10.1158/0008-5472.CAN-14-0155 24840647

[3] Nguyen YTK, Park JS, Jang JY, Kim KR, Vo TTL, Kim KW, et al. Structural and Functional Analyses of Human ChaC2 in Glutathione Metabolism. Biomolecules. 2019;10(0031). doi: 10.3390/biom10010031 31878259 PMC7022552

[4] Kamisawa T, Wood LD, Itoi T, Takaori K. Pancreatic cancer. The Lancet. 2016;388(10039):73–85. doi: 10.1016/S0140-6736(16)00141-0 26830752

[5] Kleeff J, Korc M, Apte M, La Vecchia C, Johnson CD, Biankin AV, et al. Pancreatic cancer. Nat Rev Dis Primers. 2016;2(1):1–22. doi: 10.1038/nrdp.2016.22 27158978

[6] Neoptolemos JP, Kleeff J, Michl P, Costello E, Greenhalf W, Palmer DH. Therapeutic developments in pancreatic cancer: current and future perspectives. Nat Rev Gastroenterol Hepatol. 2018;15(6):333–48. doi: 10.1038/s41575-018-0005-x 29717230

[7] Golcher H, Brunner TB, Witzigmann H, Marti L, Bechstein W-O, Bruns C, et al. Neoadjuvant chemoradiation therapy with gemcitabine/cisplatin and surgery versus immediate surgery in resectable pancreatic cancer: results of the first prospective randomized phase II trial. Strahlenther Onkol. 2015;191(1):7–16. doi: 10.1007/s00066-014-0737-7 25252602 PMC4289008

[8] Huff AL, Jaffee EM, Zaidi N. Messenger RNA vaccines for cancer immunotherapy: progress promotes promise. J Clin Invest.132(6):e156211. doi: 10.1172/JCI156211 35289317 PMC8920340

[9] Duan L-J, Wang Q, Zhang C, Yang D-X, Zhang X-Y. Potentialities and Challenges of mRNA Vaccine in Cancer Immunotherapy. Front Immunol. 2022;13. doi: 10.3389/fimmu.2022.923647 35711457 PMC9196868

[10] DiNorcia J, Lee MK, Moroziewicz DN, Winner M, Suman P, Bao F, et al. RAGE gene deletion inhibits the development and progression of ductal neoplasia and prolongs survival in a murine model of pancreatic cancer. J Gastrointest Surg. 2012;16(1):104–12; discussion 12. doi: 10.1007/s11605-011-1754-9 22052106 PMC4049447

[11] Leclerc E, Vetter SW. The role of S100 proteins and their receptor RAGE in pancreatic cancer. Biochim Biophys Acta. 2015;1852(12):2706–11. doi: 10.1016/j.bbadis.2015.09.022 26435083 PMC4643662

[12] Wu Y, Zhou Q, Guo F, Chen M, Tao X, Dong D. S100 Proteins in Pancreatic Cancer: Current Knowledge and Future Perspectives. Front Oncol. 2021;11. doi: 10.3389/fonc.2021.711180 34527585 PMC8435722

[13] Bresnick AR, Weber DJ, Zimmer DB. S100 proteins in cancer. Nat Rev Cancer. 2015;15(2):96–109. doi: 10.1038/nrc3893 25614008 PMC4369764

[14] Ikenaga N, Ohuchida K, Mizumoto K, Yu J, Fujita H, Nakata K, et al. S100A4 mRNA is a diagnostic and prognostic marker in pancreatic carcinoma. J Gastrointest Surg. 2009;13(10):1852–8. doi: 10.1007/s11605-009-0978-4 19653048

[15] Rosty C, Ueki T, Argani P, Jansen M, Yeo CJ, Cameron JL, et al. Overexpression of S100A4 in Pancreatic Ductal Adenocarcinomas Is Associated with Poor Differentiation and DNA Hypomethylation. Am J Pathol. 2002;160(1):45–50. doi: 10.1016/S0002-9440(10)64347-7 11786397 PMC1867115

[16] Logsdon CD, Simeone DM, Binkley C, Arumugam T, Greenson JK, Giordano TJ, et al. Molecular profiling of pancreatic adenocarcinoma and chronic pancreatitis identifies multiple genes differentially regulated in pancreatic cancer. Cancer Res. 2003;63(10):2649–57. 12750293

[17] Ohuchida K, Mizumoto K, Ishikawa N, Fujii K, Konomi H, Nagai E, et al. The role of S100A6 in pancreatic cancer development and its clinical implication as a diagnostic marker and therapeutic target. Clin Cancer Res. 2005;11(21):7785–93. doi: 10.1158/1078-0432.CCR-05-0714 16278400

[18] Drake CG. Combination immunotherapy approaches. Ann Oncol. 2012;23 Suppl 8(Suppl 8):viii41–6. doi: 10.1093/annonc/mds262 22918927 PMC6278955

[19] Takamatsu H, Yamamoto K-I, Tomonobu N, Murata H, Inoue Y, Yamauchi A, et al. Extracellular S100A11 Plays a Critical Role in Spread of the Fibroblast Population in Pancreatic Cancers. Oncol Res. 2019;27(6):713–27. doi: 10.3727/096504018X15433161908259 30850029 PMC7848439

[20] Andreatta M, Nielsen M. Gapped sequence alignment using artificial neural networks: application to the MHC class I system. Bioinformatics. 2016;32(4):511–7. doi: 10.1093/bioinformatics/btv639 26515819 PMC6402319

[21] Jurtz V, Paul S, Andreatta M, Marcatili P, Peters B, Nielsen M. NetMHCpan-4.0: Improved Peptide-MHC Class I Interaction Predictions Integrating Eluted Ligand and Peptide Binding Affinity Data. J Immunol. 2017;199(9):3360–8. doi: 10.4049/jimmunol.1700893 28978689 PMC5679736

[22] Kim Y, Ponomarenko J, Zhu Z, Tamang D, Wang P, Greenbaum J, et al. Immune epitope database analysis resource. Nucleic Acids Res. 2012;40(Web Server issue):W525–30. doi: 10.1093/nar/gks438 22610854 PMC3394288

[23] Lundegaard C, Nielsen M, Lund O. The validity of predicted T-cell epitopes. Trends Biotechnol. 2006;24(12):537–8. doi: 10.1016/j.tibtech.2006.10.001 17045685

[24] Moutaftsi M, Peters B, Pasquetto V, Tscharke DC, Sidney J, Bui HH, et al. A consensus epitope prediction approach identifies the breadth of murine T(CD8+)-cell responses to vaccinia virus. Nat Biotechnol. 2006;24(7):817–9. doi: 10.1038/nbt1215 16767078

[25] Nielsen M, Lundegaard C, Worning P, Lauemøller SL, Lamberth K, Buus S, et al. Reliable prediction of T-cell epitopes using neural networks with novel sequence representations. Protein Sci. 2003;12(5):1007–17. doi: 10.1110/ps.0239403 12717023 PMC2323871

[26] Peters B, Sette A. Generating quantitative models describing the sequence specificity of biological processes with the stabilized matrix method. BMC Bioinformatics. 2005;6:132. doi: 10.1186/1471-2105-6-132 15927070 PMC1173087

[27] Sidney J, Assarsson E, Moore C, Ngo S, Pinilla C, Sette A, et al. Quantitative peptide binding motifs for 19 human and mouse MHC class I molecules derived using positional scanning combinatorial peptide libraries. Immunome Res. 2008;4:2. doi: 10.1186/1745-7580-4-2 18221540 PMC2248166

[28] Vita R, Mahajan S, Overton JA, Dhanda SK, Martini S, Cantrell JR, et al. The Immune Epitope Database (IEDB): 2018 update. Nucleic Acids Res. 2019;47(D1):D339–d43. doi: 10.1093/nar/gky1006 30357391 PMC6324067

[29] Zhang Q, Wang P, Kim Y, Haste-Andersen P, Beaver J, Bourne PE, et al. Immune epitope database analysis resource (IEDB-AR). Nucleic Acids Res. 2008;36(Web Server issue):W513–8. doi: 10.1093/nar/gkn254 18515843 PMC2447801

[30] Yazdani Z, Rafiei A, Irannejad H, Yazdani M, Valadan R. Designing a novel multiepitope peptide vaccine against melanoma using immunoinformatics approach. J Biomol Struct Dyn. 2020:1–13. doi: 10.1080/07391102.2020.1846625 33226282

[31] Reynisson B, Alvarez B, Paul S, Peters B, Nielsen M. NetMHCpan-4.1 and NetMHCIIpan-4.0: improved predictions of MHC antigen presentation by concurrent motif deconvolution and integration of MS MHC eluted ligand data. Nucleic Acids Res. 2020;48(W1):W449–w54. doi: 10.1093/nar/gkaa379 32406916 PMC7319546

[32] Dhanda SK, Mahajan S, Paul S, Yan Z, Kim H, Jespersen MC, et al. IEDB-AR: immune epitope database-analysis resource in 2019. Nucleic Acids Res. 2019;47(W1):W502–w6. doi: 10.1093/nar/gkz452 31114900 PMC6602498

[33] Calis JJ, Maybeno M, Greenbaum JA, Weiskopf D, De Silva AD, Sette A, et al. Properties of MHC class I presented peptides that enhance immunogenicity. PLoS Comput Biol. 2013;9(10):e1003266. doi: 10.1371/journal.pcbi.1003266 24204222 PMC3808449

[34] Doytchinova IA, Flower DR. VaxiJen: a server for prediction of protective antigens, tumour antigens and subunit vaccines. BMC Bioinformatics. 2007;8(1):4. doi: 10.1186/1471-2105-8-4 17207271 PMC1780059

[35] Gupta S, Kapoor P, Chaudhary K, Gautam A, Kumar R, Raghava GP. In silico approach for predicting toxicity of peptides and proteins. PloS One. 2013;8(9):e73957. doi: 10.1371/journal.pone.0073957 24058508 PMC3772798

[36] Gupta S, Kapoor P, Chaudhary K, Gautam A, Kumar R, Raghava GP. Peptide toxicity prediction. Methods Mol Biol. 2015;1268:143–57. doi: 10.1007/978-1-4939-2285-7_7 25555724

[37] Holling TM, Schooten E, van Den Elsen PJ. Function and regulation of MHC class II molecules in T-lymphocytes: of mice and men. Human Immunol. 2004;65(4):282–90. doi: 10.1016/j.humimm.2004.01.005 15120183

[38] Kar T, Narsaria U, Basak S, Deb D, Castiglione F, Mueller DM, et al. A candidate multi-epitope vaccine against SARS-CoV-2. Sci Rep. 2020;10(1):10895. doi: 10.1038/s41598-020-67749-1 32616763 PMC7331818

[39] Ahmad TA, Eweida AE, El-Sayed LH. T-cell epitope mapping for the design of powerful vaccines. Vac Rep. 2016;6:13–22. doi: 10.1016/j.vacrep.2016.07.002

[40] Jespersen MC, Peters B, Nielsen M, Marcatili P. BepiPred-2.0: improving sequence-based B-cell epitope prediction using conformational epitopes. Nucleic Acids Res. 2017;45(W1):W24–w9. doi: 10.1093/nar/gkx346 28472356 PMC5570230

[41] Emini EA, Hughes JV, Perlow DS, Boger J. Induction of hepatitis A virus-neutralizing antibody by a virus-specific synthetic peptide. J Virol. 1985;55(3):836–9. doi: 10.1128/JVI.55.3.836-839.1985 2991600 PMC255070

[42] Dimitrov I, Naneva L, Doytchinova I, Bangov I. AllergenFP: allergenicity prediction by descriptor fingerprints. Bioinformatics. 2013;30(6):846–51. doi: 10.1093/bioinformatics/btt619 24167156

[43] Mahmoodi S, Amirzakaria JZ, Ghasemian A. In silico design and validation of a novel multi-epitope vaccine candidate against structural proteins of Chikungunya virus using comprehensive immunoinformatics analyses. PloS One. 2023;18(5):e0285177. doi: 10.1371/journal.pone.0285177 37146081 PMC10162528

[44] Rahman MM, Masum MHU, Talukder A, Akter R. An in silico reverse vaccinology approach to design a novel multiepitope peptide vaccine for non-small cell lung cancers. Inform Med Unlocked. 2023;37:101169. doi: 10.1016/j.imu.2023.101169

[45] Wilkins MR, Gasteiger E, Bairoch A, Sanchez JC, Williams KL, Appel RD, et al. Protein identification and analysis tools in the ExPASy server. Methods Mol Biol (Clifton, NJ). 1999;112:531–52. doi: 10.1385/1-59259-584-7:531 10027275

[46] Dimitrov I, Bangov I, Flower DR, Doytchinova I. AllerTOP v.2—a server for in silico prediction of allergens. J Mol Model. 2014;20(6):2278. doi: 10.1007/s00894-014-2278-5 24878803

[47] Dhanda SK, Vir P, Raghava GP. Designing of interferon-gamma inducing MHC class-II binders. Biol Direct. 2013;8:30. doi: 10.1186/1745-6150-8-30 24304645 PMC4235049

[48] Mitaku S, Hirokawa T. Physicochemical factors for discriminating between soluble and membrane proteins: hydrophobicity of helical segments and protein length. Protein Eng. 1999;12(11):953–7. doi: 10.1093/protein/12.11.953 10585500

[49] Mitaku S, Hirokawa T, Tsuji T. Amphiphilicity index of polar amino acids as an aid in the characterization of amino acid preference at membrane–water interfaces. Bioinformatics. 2002;18(4):608–16. doi: 10.1093/bioinformatics/18.4.608 12016058

[50] Magnan CN, Randall A, Baldi P. SOLpro: accurate sequence-based prediction of protein solubility. Bioinformatics. 2009;25(17):2200–7. doi: 10.1093/bioinformatics/btp386 19549632

[51] Hebditch M, Carballo-Amador MA, Charonis S, Curtis R, Warwicker J. Protein–Sol: a web tool for predicting protein solubility from sequence. Bioinformatics (Oxford, England). 2017;33(19):3098–100. doi: 10.1093/bioinformatics/btx345 28575391 PMC5870856

[52] Combet C, Blanchet C, Geourjon C, Deléage G. NPS@: network protein sequence analysis. Trends Biochem Sci. 2000 Mar;25(3):147–50. doi: 10.1016/s0968-0004(99)01540-6 10694887

[53] Altschul SF, Madden TL, Schäffer AA, Zhang J, Zhang Z, Miller W, et al. Gapped BLAST and PSI-BLAST: a new generation of protein database search programs. Nucleic Acids Res. 1997 Sep 1;25(17):3389–402. doi: 10.1093/nar/25.17.3389 9254694 PMC146917

[54] McGuffin LJ, Bryson K, Jones DT. The PSIPRED protein structure prediction server. Bioinformatics (Oxford, England). 2000;16(4):404–5. doi: 10.1093/bioinformatics/16.4.404 10869041

[55] Sen TZ, Jernigan RL, Garnier J, Kloczkowski A. GOR V server for protein secondary structure prediction. Bioinformatics. 2005;21(11):2787–8. doi: 10.1093/bioinformatics/bti408 15797907 PMC2553678

[56] Geourjon C, Deléage G. SOPMA: significant improvements in protein secondary structure prediction by consensus prediction from multiple alignments. Comput Appl Biosci. 1995 Dec;11(6):681–4. doi: 10.1093/bioinformatics/11.6.681 8808585

[57] Ghahremanifard P, Afzali F, Rostami A, Nayeri Z, Bambai B, Minuchehr Z. Designing a Novel Multi-epitope T Vaccine for “Targeting Protein for Xklp-2” (TPX2) in Hepatocellular Carcinoma Based on Immunoinformatics Approach. Int J Pept Res Ther. 2020;26(2):1127–36. doi: 10.1007/s10989-019-09915-2

[58] Masum MHU, Ferdous J, Lokman S, Siddiki AZ. Designing of a multiepitope-based chimeric vaccine against dengue virus serotype 3 (DENV-3) through next generation reverse vaccinology approaches. Inform Med Unlocked. 2024;44:101422. doi: 10.1016/j.imu.2023.10142

[59] Wu S, Skolnick J, Zhang Y. Ab initio modeling of small proteins by iterative TASSER simulations. BMC Biol. 2007 May 8;5:17. doi: 10.1186/1741-7007-5-17 17488521 PMC1878469

[60] Zhang Y. I-TASSER server for protein 3D structure prediction. BMC bioinformatics. 2008;9(1):40. doi: 10.1186/1471-2105-9-40 18215316 PMC2245901

[61] Ko J, Park H, Heo L, Seok C. GalaxyWEB server for protein structure prediction and refinement. Nucleic Acids Res. 2012 Jul;40(Web Server issue):W294–7. doi: 10.1093/nar/gks493 22649060 PMC3394311

[62] Laskowski RA, MacArthur MW, Moss DS, Thornton JM. PROCHECK: a program to check the stereochemical quality of protein structures. J Appl Crystallogr. 1993;26(2):283–91. doi: 10.1107/S0021889892009944

[63] Laskowski RA, MacArthur MW, Thornton JM. PROCHECK: validation of protein-structure coordinates. Int Tab Crystallogr. p. 684–7. doi: 10.1107/97809553602060000882

[64] Laskowski RA, Rullmannn JA, MacArthur MW, Kaptein R, Thornton JM. AQUA and PROCHECK-NMR: programs for checking the quality of protein structures solved by NMR. J Biomol NMR. 1996 Dec;8(4):477–86. doi: 10.1007/BF00228148 9008363

[65] Morris AL, MacArthur MW, Hutchinson EG, Thornton JM. Stereochemical quality of protein structure coordinates. Proteins. 1992;12(4):345–64. doi: 10.1002/prot.340120407 1579569

[66] Sippl MJ. Recognition of errors in three-dimensional structures of proteins. Proteins. 1993;17(4):355–62. doi: 10.1002/prot.340170404 8108378

[67] Wiederstein M, Sippl MJ. ProSA-web: interactive web service for the recognition of errors in three-dimensional structures of proteins. Nucleic Acids Res. 2007 Jul;35(Web Server issue):W407–10. doi: 10.1093/nar/gkm290 17517781 PMC1933241

[68] Desta IT, Porter KA, Xia B, Kozakov D, Vajda S. Performance and Its Limits in Rigid Body Protein-Protein Docking. Structure (London, England: 1993). 2020;28(9):1071–81.e3. doi: 10.1016/j.str.2020.06.006 32649857 PMC7484347

[69] Kozakov D, Beglov D, Bohnuud T, Mottarella SE, Xia B, Hall DR, et al. How good is automated protein docking? Proteins. 2013;81(12):2159–66. doi: 10.1002/prot.24403 23996272 PMC3934018

[70] Kozakov D, Hall DR, Xia B, Porter KA, Padhorny D, Yueh C, et al. The ClusPro web server for protein-protein docking. Nat Protoc. 2017 Feb;12(2):255–278. doi: 10.1038/nprot.2016.169 28079879 PMC5540229

[71] Vajda S, Yueh C, Beglov D, Bohnuud T, Mottarella SE, Xia B, et al. New additions to the ClusPro server motivated by CAPRI. Proteins. 2017;85(3):435–44. doi: 10.1002/prot.25219 27936493 PMC5313348

[72] Comeau S, Vajda S, Camacho C. Performance of the first protein docking server ClusPro in CAPRI Rounds 3–5. Proteins. 2005;60:239–44. doi: 10.1002/prot.20564 15981265

[73] Fransen F, Stenger RM, Poelen MC, van Dijken HH, Kuipers B, Boog CJ, et al. Differential effect of TLR2 and TLR4 on the immune response after immunization with a vaccine against Neisseria meningitidis or Bordetella pertussis. PLoS One. 2010 Dec 23;5(12):e15692. doi: 10.1371/journal.pone.0015692 21203418 PMC3009743

[74] Yang JX, Tseng JC, Yu GY, Luo Y, Huang CF, Hong YR, et al. Recent Advances in the Development of Toll-like Receptor Agonist-Based Vaccine Adjuvants for Infectious Diseases. Pharmaceutics. 2022;14(2). doi: 10.3390/pharmaceutics14020423 35214155 PMC8878135

[75] Chen F, Sun H, Wang J, Zhu F, Liu H, Wang Z, et al. Assessing the performance of MM/PBSA and MM/GBSA methods. 8. Predicting binding free energies and poses of protein-RNA complexes. RNA. 2018 Sep;24(9):1183–1194. doi: 10.1261/rna.065896.118 29930024 PMC6097651

[76] Hou T, Wang J, Li Y, Wang W. Assessing the performance of the MM/PBSA and MM/GBSA methods. 1. The accuracy of binding free energy calculations based on molecular dynamics simulations. J Chem Inf Model. 2011 Jan 24;51(1):69–82. doi: 10.1021/ci100275a 21117705 PMC3029230

[77] Sun H, Li Y, Tian S, Xu L, Hou T. Assessing the performance of MM/PBSA and MM/GBSA methods. 4. Accuracies of MM/PBSA and MM/GBSA methodologies evaluated by various simulation protocols using PDBbind data set. Phys Chem Chem Phys. 2014 Aug 21;16(31):16719–29. doi: 10.1039/c4cp01388c 24999761

[78] Weng G, Wang E, Wang Z, Liu H, Zhu F, Li D, et al. HawkDock: a web server to predict and analyze the protein–protein complex based on computational docking and MM/GBSA. Nucleic Acids Res. 2019 Jul 2;47(W1):W322–W330. doi: 10.1093/nar/gkz397 31106357 PMC6602443

[79] Ponomarenko J, Bui HH, Li W, Fusseder N, Bourne PE, Sette A, et al. ElliPro: a new structure-based tool for the prediction of antibody epitopes. BMC Bioinformatics. 2008 Dec 2;9:514. doi: 10.1186/1471-2105-9-514 19055730 PMC2607291

[80] Bibi S, Ullah I, Zhu B, Adnan M, Liaqat R, Kong W-B, et al. In silico analysis of epitope-based vaccine candidate against tuberculosis using reverse vaccinology. Sci Rep. 2021 Jan 13;11(1):1249. doi: 10.1038/s41598-020-80899-6 33441913 PMC7807040

[81] Rapin N, Lund O, Bernaschi M, Castiglione F. Computational immunology meets bioinformatics: the use of prediction tools for molecular binding in the simulation of the immune system. PLoS One. 2010 Apr 16;5(4):e9862. doi: 10.1371/journal.pone.0009862 20419125 PMC2855701

[82] Sanami S, Zandi M, Pourhossein B, Mobini GR, Safaei M, Abed A, et al. Design of a multi-epitope vaccine against SARS-CoV-2 using immunoinformatics approach. Int J Biol Macromol. 2020 Dec 1;164:871–883. doi: 10.1016/j.ijbiomac.2020.07.117 32682041 PMC7362859

[83] Gruber AR, Lorenz R, Bernhart SH, Neuböck R, Hofacker IL. The Vienna RNA Websuite. Nucleic Acids Res. 2008 Jul 1;36(Web Server issue):W70–4. doi: 10.1093/nar/gkn188 18424795 PMC2447809

[84] Lorenz R, Bernhart SH, Höner zu Siederdissen C, Tafer H, Flamm C, Stadler PF, et al. ViennaRNA Package 2.0. Algorithms Mol Biol. 2011 Nov 24;6:26. doi: 10.1186/1748-7188-6-26 22115189 PMC3319429

[85] Motamedi H, Alvandi A, Fathollahi M, Ari MM, Moradi S, Moradi J, et al. In silico designing and immunoinformatics analysis of a novel peptide vaccine against metallo-beta-lactamase (VIM and IMP) variants. PLoS One. 2023 Jul 20;18(7):e0275237. doi: 10.1371/journal.pone.0275237 37471423 PMC10358925

[86] Zhao Y, Wang J, Zeng C, Xiao Y. Evaluation of RNA secondary structure prediction for both base-pairing and topology. Biophys Rep. 2018;4(3):123–32. doi: 10.1007/s41048-018-0058-y

[87] Abass OA, Timofeev VI, Sarkar B, Onobun DO, Ogunsola SO, Aiyenuro AE, et al. Immunoinformatics analysis to design novel epitope based vaccine candidate targeting the glycoprotein and nucleoprotein of Lassa mammarenavirus (LASMV) using strains from Nigeria. J Biomol Struct Dyn. 2022 Oct;40(16):7283–7302. doi: 10.1080/07391102.2021.1896387 33719908

[88] Araf Y, Moin AT, Timofeev VI, Faruqui NA, Saiara SA, Ahmed N, et al. Immunoinformatic Design of a Multivalent Peptide Vaccine Against Mucormycosis: Targeting FTR1 Protein of Major Causative Fungi. Front Immunol. 2022 May 26;13:863234. doi: 10.3389/fimmu.2022.863234 35720422 PMC9204303

[89] Hamasaki-Katagiri N, Lin BC, Simon J, Hunt RC, Schiller T, Russek-Cohen E, et al. The importance of mRNA structure in determining the pathogenicity of synonymous and non-synonymous mutations in haemophilia. Haemophilia. 2017 Jan;23(1):e8–e17. doi: 10.1111/hae.13107 27933712 PMC5226872

[90] Mugunthan SP, Harish MC. Multi-epitope-Based Vaccine Designed by Targeting Cytoadherence Proteins of Mycoplasma gallisepticum. ACS Omega. 2021 May 17;6(21):13742–13755. doi: 10.1021/acsomega.1c01032 34095666 PMC8173551

[91] Liu J, Fu M, Wang M, Wan D, Wei Y, Wei X. Cancer vaccines as promising immuno-therapeutics: platforms and current progress. J Hematol Oncol. 2022 Mar 18;15(1):28. doi: 10.1186/s13045-022-01247-x 35303904 PMC8931585

[92] Hoover HC, Jr., Surdyke MG, Dangel RB, Peters LC, Hanna MG Jr. Prospectively randomized trial of adjuvant active-specific immunotherapy for human colorectal cancer. Cancer. 1985 Mar 15;55(6):1236–43. doi: 10.1002/1097-0142(19850315)55:6&lt;1236::aid-cncr2820550616&gt;3.0.co;2-# 3882219

[93] van der Bruggen P, Traversari C, Chomez P, Lurquin C, De Plaen E, Van den Eynde B, et al. A gene encoding an antigen recognized by cytolytic T lymphocytes on a human melanoma. Science. 1991 Dec 13;254(5038):1643–7. doi: 10.1126/science.1840703 1840703

[94] Miao L, Zhang Y, Huang L. mRNA vaccine for cancer immunotherapy. Mol Cancer. 2021 Feb 25;20(1):41. doi: 10.1186/s12943-021-01335-5 33632261 PMC7905014

[95] Saxena M, van der Burg SH, Melief CJM, Bhardwaj N. Therapeutic cancer vaccines. Nat Rev Cancer. 2021 Jun;21(6):360–378. doi: 10.1038/s41568-021-00346-0 33907315

[96] Mohammadi Y, Nezafat N, Negahdaripour M, Eskandari S, Zamani M. In silico design and evaluation of a novel mRNA vaccine against BK virus: a reverse vaccinology approach. Immunol Res. 2023 Jun;71(3):422–441. doi: 10.1007/s12026-022-09351-3 36580228 PMC9797904

[97] Pardi N, Hogan MJ, Porter FW, Weissman D. mRNA vaccines—a new era in vaccinology. Nat Rev Drug Discov. 2018 Apr;17(4):261–279. doi: 10.1038/nrd.2017.243 29326426 PMC5906799

[98] Guan S, Rosenecker J. Nanotechnologies in delivery of mRNA therapeutics using nonviral vector-based delivery systems. Gene Ther. 2017 Mar;24(3):133–143. doi: 10.1038/gt.2017.5 28094775

[99] Karikó K, Muramatsu H, Welsh FA, Ludwig J, Kato H, Akira S, et al. Incorporation of pseudouridine into mRNA yields superior nonimmunogenic vector with increased translational capacity and biological stability. Mol Ther. 2008 Nov;16(11):1833–40. doi: 10.1038/mt.2008.200 18797453 PMC2775451

[100] Kauffman KJ, Webber MJ, Anderson DG. Materials for non-viral intracellular delivery of messenger RNA therapeutics. J Control Release. 2016 Oct 28;240:227–234. doi: 10.1016/j.jconrel.2015.12.032 26718856

[101] Thess A, Grund S, Mui BL, Hope MJ, Baumhof P, Fotin-Mleczek M, et al. Sequence-engineered mRNA Without Chemical Nucleoside Modifications Enables an Effective Protein Therapy in Large Animals. Mol Ther. 2015 Sep;23(9):1456–64. doi: 10.1038/mt.2015.103 26050989 PMC4817881

[102] Karikó K, Muramatsu H, Ludwig J, Weissman D. Generating the optimal mRNA for therapy: HPLC purification eliminates immune activation and improves translation of nucleoside-modified, protein-encoding mRNA. Nucleic Acids Res. 2011 Nov;39(21):e142. doi: 10.1093/nar/gkr695 21890902 PMC3241667

[103] Sahin U, Karikó K, Türeci Ö. mRNA-based therapeutics—developing a new class of drugs. Nat Rev Drug Discov. 2014 Oct;13(10):759–80. doi: 10.1038/nrd4278 25233993

[104] Parvizpour S, Pourseif MM, Razmara J, Rafi MA, Omidi Y. Epitope-based vaccine design: a comprehensive overview of bioinformatics approaches. Drug Discov Today. 2020 Jun;25(6):1034–1042. doi: 10.1016/j.drudis.2020.03.006 32205198

[105] Ahammad I, Lira SS. Designing a novel mRNA vaccine against SARS-CoV-2: An immunoinformatics approach. Int J Biol Macromol. 2020 Nov 1;162:820–837. doi: 10.1016/j.ijbiomac.2020.06.213 32599237 PMC7319648

[106] Fotin-Mleczek M, Duchardt KM, Lorenz C, Pfeiffer R, Ojkić-Zrna S, Probst J, et al. Messenger RNA-based vaccines with dual activity induce balanced TLR-7 dependent adaptive immune responses and provide antitumor activity. J Immunother. 2011 Jan;34(1):1–15. doi: 10.1097/CJI.0b013e3181f7dbe8 21150709

[107] Schlake T, Thess A, Fotin-Mleczek M, Kallen KJ. Developing mRNA-vaccine technologies. RNA Biol. 2012 Nov;9(11):1319–30. doi: 10.4161/rna.22269 23064118 PMC3597572

[108] Geall AJ, Verma A, Otten GR, Shaw CA, Hekele A, Banerjee K, et al. Nonviral delivery of self-amplifying RNA vaccines. Proc Natl Acad Sci U S A. 2012 Sep 4;109(36):14604–9. doi: 10.1073/pnas.1209367109 22908294 PMC3437863

[109] Pardi N, Hogan MJ, Pelc RS, Muramatsu H, Andersen H, DeMaso CR, et al. Zika virus protection by a single low-dose nucleoside-modified mRNA vaccination. Nature. 2017 Mar 9;543(7644):248–251. doi: 10.1038/nature21428 28151488 PMC5344708

[110] Petsch B, Schnee M, Vogel AB, Lange E, Hoffmann B, Voss D, et al. Protective efficacy of in vitro synthesized, specific mRNA vaccines against influenza A virus infection. Nat Biotechnol. 2012 Dec;30(12):1210–6. doi: 10.1038/nbt.2436 23159882

[111] Anand P, Stahel VPJPsis. The safety of Covid-19 mRNA vaccines: a review. Patient Saf Surg. 2021 May 1;15(1):20. doi: 10.1186/s13037-021-00291-9 33933145 PMC8087878

[112] Negahdaripour M, Shafiekhani M, Moezzi SMI, Amiri S, Rasekh S, Bagheri A, et al. Administration of COVID-19 vaccines in immunocompromised patients. I Int Immunopharmacol. 2021 Oct;99:108021. doi: 10.1016/j.intimp.2021.108021 34352567 PMC8316069

[113] Press release. NobelPrize.org. Nobel Prize Outreach AB 2024. 2023 Oct 02 [Cited 2024 Jan 22]. Available from: https://www.nobelprize.org/prizes/medicine/2023/press-release/.

[114] Pollard C, De Koker S, Saelens X, Vanham G, Grooten J. Challenges and advances towards the rational design of mRNA vaccines. Trends Mol Med. 2013 Dec;19(12):705–13. doi: 10.1016/j.molmed.2013.09.002 24138818

[115] Van Nuffel AM, Wilgenhof S, Thielemans K, Bonehill A. Overcoming HLA restriction in clinical trials: Immune monitoring of mRNA-loaded DC therapy. Oncoimmunology. 2012 Nov 1;1(8):1392–1394. doi: 10.4161/onci.20926 23243604 PMC3518513

[116] Pardi N, Hogan MJ, Weissman D. Recent advances in mRNA vaccine technology. Curr Opin Immunol. 2020 Aug;65:14–20. doi: 10.1016/j.coi.2020.01.008 32244193

[117] Brito LA, Kommareddy S, Maione D, Uematsu Y, Giovani C, Berlanda Scorza F, et al. Self-amplifying mRNA vaccines. Adv Genet. 2015;89:179–233. doi: 10.1016/bs.adgen.2014.10.005 25620012

[118] Rojas LA, Sethna Z, Soares KC, Olcese C, Pang N, Patterson E, et al. Personalized RNA neoantigen vaccines stimulate T cells in pancreatic cancer. Nature. 2023 Jun;618(7963):144–150. doi: 10.1038/s41586-023-06063-y 37165196 PMC10171177

[119] Comber JD, Philip R. MHC class I antigen presentation and implications for developing a new generation of therapeutic vaccines. Ther Adv Vaccines. 2014 May;2(3):77–89. doi: 10.1177/2051013614525375 24790732 PMC3991156

[120] Duffy EB, Drake JR, Harton JA. Evolving Insights for MHC Class II Antigen Processing and Presentation in Health and Disease. Curr Pharmacol Rep. 2017;3(5):213–20. doi: 10.1007/s40495-017-0097-y

[121] Blum JS, Wearsch PA, Cresswell P. Pathways of antigen processing. Annu Rev Immunol. 2013;31:443–73. doi: 10.1146/annurev-immunol-032712-095910 23298205 PMC4026165

[122] Roche PA, Furuta K. The ins and outs of MHC class II-mediated antigen processing and presentation. Nat Rev Immunol. 2015 Apr;15(4):203–16. doi: 10.1038/nri3818 25720354 PMC6314495

[123] Unanue ER, Turk V, Neefjes J. Variations in MHC Class II Antigen Processing and Presentation in Health and Disease. Annu Rev Immunol. 2016 May 20;34:265–97. doi: 10.1146/annurev-immunol-041015-055420 26907214

[124] Russell SL, Lamprecht DA, Mandizvo T, Jones TT, Naidoo V, Addicott KW, et al. Compromised Metabolic Reprogramming Is an Early Indicator of CD8(+) T Cell Dysfunction during Chronic Mycobacterium tuberculosis Infection. Cell Rep. 2019 Dec 10;29(11):3564–3579.e5. doi: 10.1016/j.celrep.2019.11.034 31825836 PMC6915325

[125] Patankar YR, Sutiwisesak R, Boyce S, Lai R, Lindestam Arlehamn CS, Sette A, et al. Limited recognition of Mycobacterium tuberculosis-infected macrophages by polyclonal CD4 and CD8 T cells from the lungs of infected mice. Mucosal Immunol. 2020 Jan;13(1):140–148. doi: 10.1038/s41385-019-0217-6 31636345 PMC7161428

[126] Lu LL, Suscovich TJ, Fortune SM, Alter G. Beyond binding: antibody effector functions in infectious diseases. Nat Rev Immunol. 2018 Jan;18(1):46–61. doi: 10.1038/nri.2017.106 29063907 PMC6369690

[127] EL-Manzalawy Y, Dobbs D, Honavar V. Predicting linear B-cell epitopes using string kernels. J Mol Recognit. 2008 Jul-Aug;21(4):243–55. doi: 10.1002/jmr.893 18496882 PMC2683948

[128] Krocova Z, Plzakova L, Pavkova I, Kubelkova K, Macela A, Ozanic M, et al. The role of B cells in an early immune response to Mycobacterium bovis. Microb Pathog. 2020 Mar;140:103937. doi: 10.1016/j.micpath.2019.103937 31862393

[129] Khatoon N, Pandey RK, Prajapati VK. Exploring Leishmania secretory proteins to design B and T cell multi-epitope subunit vaccine using immunoinformatics approach. Sci Rep. 2017 Aug 15;7(1):8285. doi: 10.1038/s41598-017-08842-w 28811600 PMC5557753

[130] Meza B, Ascencio F, Sierra-Beltrán AP, Torres J, Angulo C. A novel design of a multi-antigenic, multistage and multi-epitope vaccine against Helicobacter pylori: An in silico approach. Infect Genet Evol. 2017 Apr;49:309–317. doi: 10.1016/j.meegid.2017.02.007 28185986

[131] Al Tbeishat HJSR. Novel In Silico mRNA vaccine design exploiting proteins of M. tuberculosis that modulates host immune responses by inducing epigenetic modifications. Sci Rep. 2022 Mar 17;12(1):4645. doi: 10.1038/s41598-022-08506-4 35301360 PMC8929471

