# Supplementary figures and images for: An mRNA vaccine for pancreatic cancer designed by applying in silico immunoinformatics and reverse vaccinology approaches
Source: PLoS One. 2024 Jul 8;19(7):e0305413. doi: 10.1371/journal.pone.0305413 (PMC11230540; doi:10.1371/journal.pone.0305413)

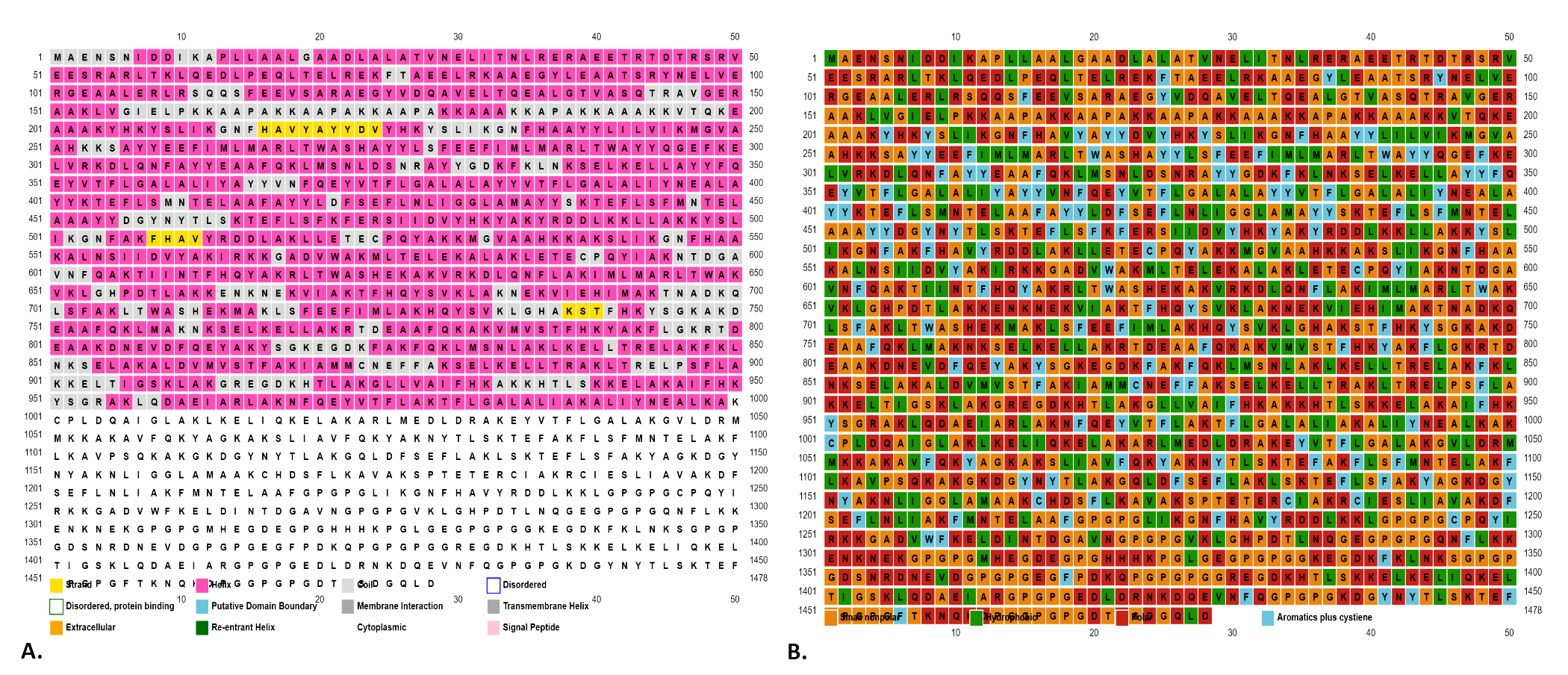

Supplement: S1 Fig — The secondary structure of the vaccine was predicted by the PSIPRED server, with the features (A) and types (B) of amino acids of the vaccine. In the sequence, among 1479 amino acids, where most of the amino acids were in the coil structure (grey), lesser in the helix structure (pink color), and least in the strand (yellow color) (A). The different types of amino acids in the sequence have been exhibited: the small nonpolar amino acids were predominant (orange), the hydrophobic amino acids (green) and the polar amino acids (red) were less prominent, and the aromatic plus cysteine residues (sky blue) were least prominent (B). (TIF) [file pone.0305413.s001.tif]

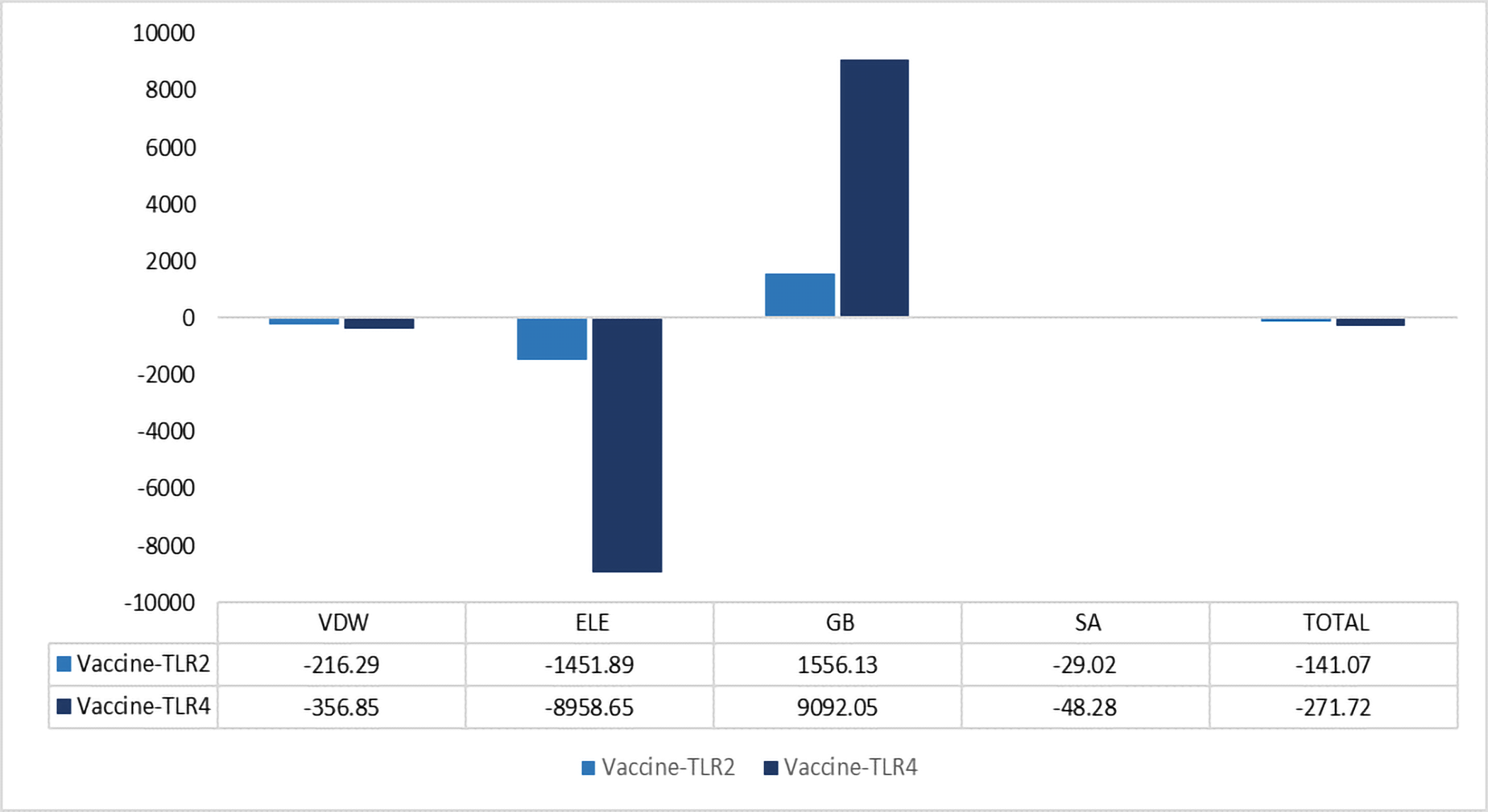

Supplement: S2 Fig — (TIF) [file pone.0305413.s002.tif]

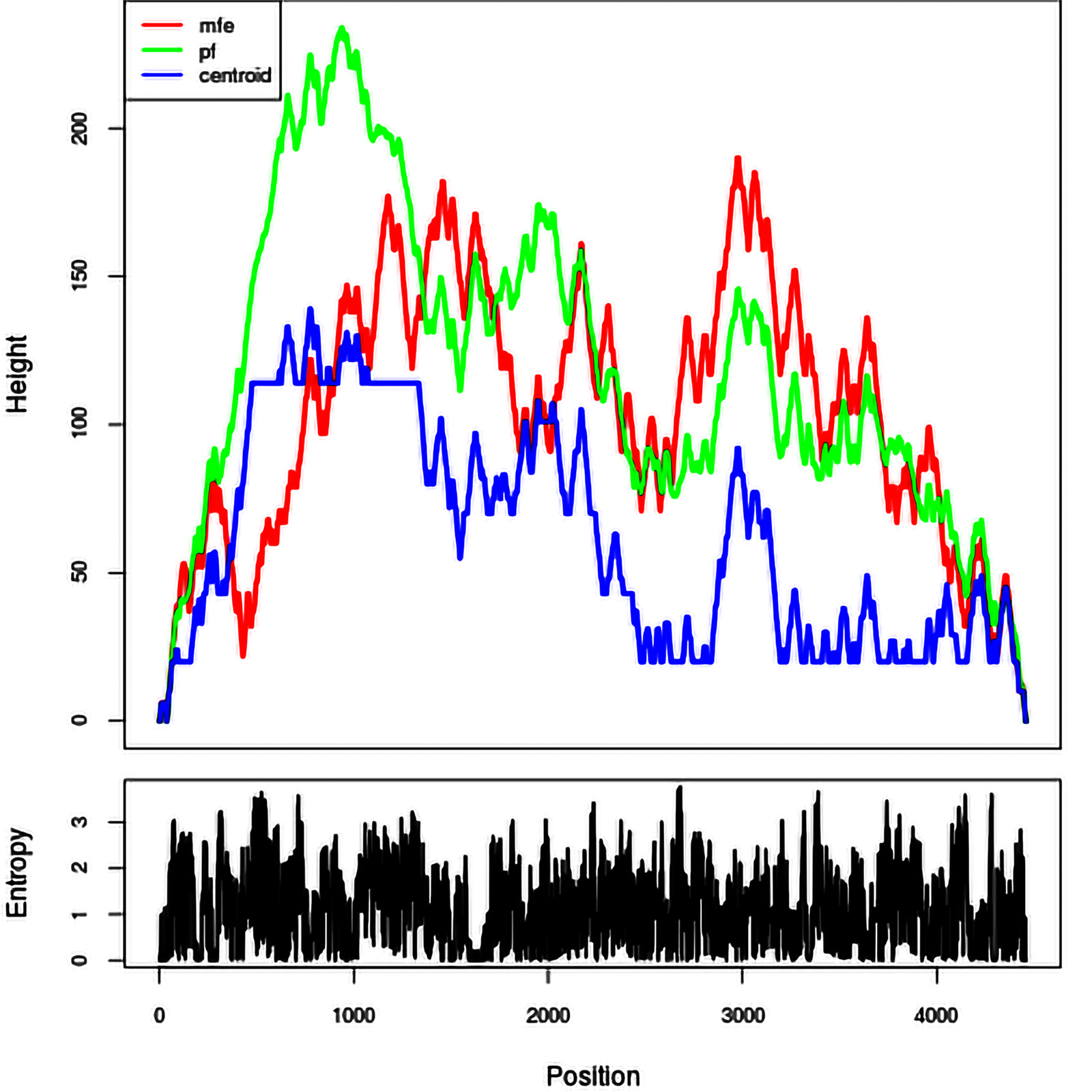

Supplement: S3 Fig — (TIF) [file pone.0305413.s003.tif]
