# Supplementary material for: An mRNA vaccine for pancreatic cancer designed by applying in silico immunoinformatics and reverse vaccinology approaches
Source: PLoS One. 2024 Jul 8;19(7):e0305413. doi: 10.1371/journal.pone.0305413 (PMC11230540; doi:10.1371/journal.pone.0305413)
Supplement: S1 Table — (DOCX) [file pone.0305413.s004.docx]

**S1 Table.** The interactions between the vaccine and TLRs.

| Complex | Weighted Score | | Interactions between the vaccine and TLRs | | | |
| --- | --- | --- | --- | --- | --- | --- |
|  | Center | Lowest energy | Salt  bridges | Disulphide  bonds | Hydrogen  bonds | Non-bonded  contacts |
| Vaccine-TLR-2 | -1031.7 | -1031.7 | 7 | - | 24 | 251 |
| Vaccine-TLR-4 | -1069.8 | -1313.6 | 48 | - | 17 | 446 |
