# Supplementary material for: An mRNA vaccine for pancreatic cancer designed by applying in silico immunoinformatics and reverse vaccinology approaches
Source: PLoS One. 2024 Jul 8;19(7):e0305413. doi: 10.1371/journal.pone.0305413 (PMC11230540; doi:10.1371/journal.pone.0305413)
Supplement: S2 Table — (DOCX) [file pone.0305413.s005.docx]

**S2 Table.** The discontinuous B-cell epitopes of the vaccine by Ellipro server.

| No. | Residues | Number of residues | Score |
| --- | --- | --- | --- |
| 1 | A:T197, A:Q198, A:K199, A:E200, A:A201, A:A202, A:A203, A:K204, A:Y205, A:H206, A:K207, A:Y208, A:S209, A:L210, A:I211, A:K212, A:G213, A:N214, A:F215, A:H216, A:A217, A:V218, A:Y219, A:A220, A:Y221, A:Y222, A:D223, A:V224, A:Y225, A:H226, A:K227, A:Y228, A:S229, A:L230, A:I231, A:K232, A:G233, A:N234, A:F235, A:H236, A:A237, A:A238, A:Y239, A:Y240, A:L241, A:I242, A:L243, A:K246, A:Y257, A:E260, A:I262, A:M263, A:L264, A:M265, A:A266, A:R267, A:L268, A:T269, A:W270, A:A271, A:S272, A:H273, A:A274, A:Y275, A:Y276, A:L277, A:S278, A:F279, A:E280, A:E281, A:F282, A:I283, A:M284, A:L285, A:M286, A:A287, A:R288, A:L289, A:T290, A:W291, A:A292, A:Y293 | 82 | 0.792 |
| 2 | A:L1263, A:T1267, A:G1269, A:A1270, A:V1271, A:N1272, A:G1273, A:P1274, A:G1275, A:P1276, A:G1277, A:V1278, A:K1279, A:L1280, A:G1281, A:H1282, A:P1283, A:D1284, A:T1285, A:L1286, A:N1287, A:Q1288, A:G1289, A:E1290, A:G1291, A:P1292, A:G1293, A:P1294, A:G1295, A:Q1296, A:N1297, A:F1298, A:L1299, A:K1300, A:K1301, A:E1302, A:N1303, A:K1304, A:N1305, A:E1306, A:K1307, A:G1308, A:P1309, A:G1310, A:P1311, A:G1312, A:M1313, A:H1314, A:E1315, A:G1316, A:D1317, A:E1318, A:G1319, A:P1320, A:G1321, A:H1322, A:H1323, A:H1324, A:K1325, A:P1326, A:G1327, A:L1328, A:G1329, A:E1330, A:G1331, A:P1332, A:G1333, A:P1334, A:G1335, A:G1336, A:K1337, A:E1338, A:G1339, A:D1340, A:K1341, A:F1342, A:K1343, A:L1344, A:N1345, A:K1346, A:S1347, A:G1348, A:P1349, A:G1350, A:P1351, A:G1352, A:D1353, A:S1354, A:N1355, A:R1356, A:D1357, A:N1358, A:E1359, A:V1360, A:D1361, A:G1362, A:P1363, A:G1364, A:P1365, A:G1366, A:E1367, A:G1368, A:F1369, A:P1370, A:D1371, A:K1372, A:Q1373, A:P1374, A:G1375, A:P1376, A:G1377, A:P1378, A:G1379, A:G1380, A:R1381, A:E1382, A:G1383, A:D1384, A:K1385, A:H1386, A:T1387, A:L1388, A:S1389, A:K1390, A:K1391, A:E1392, A:L1393, A:K1394, A:E1395, A:L1396, A:I1397, A:Q1398, A:K1399, A:E1400, A:L1401, A:T1402, A:I1403, A:G1404, A:S1405, A:K1406, A:L1407, A:Q1408, A:D1409, A:A1410, A:I1412, A:A1413, A:R1414, A:G1415, A:P1416, A:G1417, A:P1418, A:G1419, A:E1420, A:D1421, A:L1422, A:D1423, A:R1424, A:N1425, A:K1426, A:D1427, A:Q1428, A:E1429, A:V1430, A:N1431, A:F1432, A:Q1433, A:G1434, A:P1435, A:G1436, A:P1437, A:G1438, A:K1439, A:D1440, A:G1441, A:Y1442, A:N1443, A:Y1444, A:T1445, A:L1446, A:S1447, A:K1448, A:T1449, A:E1450, A:F1451, A:G1452, A:P1453, A:G1454, A:P1455, A:G1456, A:F1457, A:T1458, A:K1459, A:N1460, A:Q1461, A:K1462, A:D1463, A:P1464, A:G1465, A:G1466, A:P1467, A:G1468, A:P1469, A:G1470, A:D1471, A:T1472, A:N1473, A:S1474, A:D1475, A:G1476, A:Q1477, A:L1478 | 211 | 0.782 |
| 3 | A:L25, A:V28, A:N29, A:L31, A:I32, A:T33, A:N34, A:L35, A:R36, A:E37, A:R38, A:A39, A:E40, A:E41, A:T42, A:R43, A:T44, A:D45, A:T46, A:R47, A:S48, A:R49, A:V50, A:E51, A:E52, A:S53, A:R54, A:A55, A:R56, A:L57, A:T58, A:K59, A:L60, A:Q61, A:E62, A:D63, A:L64, A:P65, A:E66, A:Q67, A:L68, A:T69, A:E70, A:L71, A:R72, A:E73, A:K74, A:F75, A:T76, A:A77, A:E78, A:E79, A:L80, A:R81, A:K82, A:A83, A:A84, A:E85, A:G86, A:Y87, A:L88, A:E89, A:A90, A:A91, A:T92, A:S93, A:R94, A:Y95, A:N96, A:E97, A:L98, A:V99, A:E100, A:R101, A:G102, A:E103, A:A104, A:A105, A:L106, A:E107, A:R108, A:L109, A:R110, A:S111, A:Q112, A:Q113, A:S114, A:F115, A:E116, A:E117, A:V118, A:S119, A:A120, A:R121, A:A122, A:E123, A:G124, A:Y125, A:V126, A:D127, A:A129, A:V130, A:E131, A:T144, A:A146, A:V147, A:G148, A:E149, A:R150, A:A151, A:A152, A:K153, A:L154, A:V155, A:G156, A:I157, A:E158, A:L159, A:P160, A:K161, A:K162, A:A163, A:A164, A:P165, A:A166, A:K167, A:K168, A:A169, A:A170, A:P171, A:A172, A:K173, A:K174, A:A175, A:A176, A:A178 | 136 | 0.732 |
| 4 | A:G567, A:A568, A:D569, A:A572, A:M573, A:T575, A:E576, A:L577, A:E578, A:K579, A:A580, A:L581, A:A582, A:K583, A:L584, A:E585, A:T586, A:E587, A:C588, A:P589, A:Q590, A:Y591, A:I592, A:A593, A:K594, A:N595, A:T596, A:D597, A:G598, A:A599, A:V600, A:N601, A:F602, A:Q603, A:A604, A:K605, A:T606, A:I607, A:I608, A:N609, A:T610, A:F611, A:H612, A:Q613, A:Y614, A:K616, A:R617, A:W620, A:K630, A:D631, A:Q633, A:N634, A:F635, A:L636, A:A637, A:K638, A:I639, A:M640, A:L641, A:M642, A:A643, A:R644, A:L645, A:T646, A:W647, A:A648, A:K649, A:V650, A:K651, A:L652, A:G653, A:H654, A:P655, A:D656, A:T657, A:L658, A:A659, A:K660, A:K661, A:E662, A:N663, A:K664, A:N665, A:E666, A:K667, A:V668, A:I669, A:A670, A:K671, A:F673, A:H674, A:A703, A:K704, A:L705, A:T706, A:W707, A:A708, A:S709, A:H710, A:E711, A:K712, A:M713, A:A714, A:K715, A:L716, A:S717, A:F718, A:E719, A:H741, A:K742, A:Y743, A:S744, A:G745, A:K746, A:A747, A:K748, A:D749, A:E750, A:A751, A:A752, A:F753, A:Q754, A:K755, A:L756, A:M757, A:A758, A:K759, A:N760, A:K761, A:S762, A:E763, A:L764, A:K765, A:E766, A:L767, A:L768, A:A769, A:K770, A:R771, A:T772, A:D773, A:E774, A:A775, A:A776, A:F777, A:Q778, A:A927, A:H930, A:K931, A:A932, A:K933, A:K934, A:H935, A:T936, A:L937, A:S938, A:K939, A:K940, A:E941, A:L942, A:A943, A:K944, A:A945, A:I946, A:F947, A:H948, A:K949, A:Y950, A:S951, A:G952, A:R953, A:A954, A:K955, A:L956, A:Q957, A:D958, A:A959, A:E960, A:I961, A:A962, A:R963, A:L964, A:A965, A:K966, A:N967, A:F968, A:Q969, A:E970, A:Y971, A:V972, A:T973, A:F974, A:L975, A:A976, A:K977, A:T978 | 196 | 0.73 |
| 5 | A:Y815, A:S816, A:G817, A:K818, A:E819, A:G820, A:D821, A:K822, A:F823, A:A824, A:K825, A:F826, A:Q827, A:K828, A:A869, A:M870, A:M871, A:C872, A:N873, A:E874, A:F875, A:F876, A:A877, A:K878, A:S879, A:E880, A:L881, A:K882, A:E883, A:L884, A:L885, A:T886, A:R887, A:A888, A:K889, A:L890, A:T891 | 37 | 0.696 |
| 6 | A:L443, A:S444, A:F445, A:N447, A:T448, A:E449, A:L450, A:A451, A:A452, A:A453, A:Y454, A:Y455, A:D456, A:G457, A:Y458, A:N459, A:Y460, A:T461, A:F505, A:A506, A:K507, A:H509, A:A510, A:V511, A:Y512, A:D514, A:D515, A:K518, A:L519, A:T522 | 30 | 0.621 |
| 7 | A:M1, A:A2, A:E3, A:N4, A:S5, A:N6, A:I7, A:E994, A:L996, A:K997, A:A998, A:K999, A:C1000, A:P1001, A:L1002, A:D1003, A:Q1004, A:A1005, A:I1006, A:G1007, A:L1008, A:A1009, A:K1010, A:L1011, A:K1012, A:E1013, A:L1014, A:I1015, A:Q1016, A:K1017, A:E1018, A:L1019, A:Q1072, A:K1076, A:N1077, A:Y1078, A:T1079, A:L1080, A:S1081, A:K1082, A:T1083, A:F1085, A:A1086 | 43 | 0.603 |
| 8 | A:A539, A:K540, A:S541, A:L542, A:I543, A:N546, A:H548 | 7 | 0.555 |
| 9 | A:V533, A:H536, A:K537, A:K538 | 4 | 0.535 |
